# Diagnostic and prognostic value of galactose-deficient IgA1 in patients with IgA nephropathy: an updated systematic review with meta-analysis

**DOI:** 10.3389/fimmu.2023.1209394

**Published:** 2023-08-21

**Authors:** Qin Zeng, Wen-Ru Wang, Yi-Han Li, Ying Liang, Xin-Hui Wang, Lei Yan, Ren-Huan Yu

**Affiliations:** Department of Nephrology, Xiyuan Hospital of China Academy of Chinese Medical Sciences, Beijing, China

**Keywords:** immunoglobulin A nephropathy, galactose-deficient IgA1, systematic review and meta-analysis, biomarker, noninvasive prognosis

## Abstract

**Objectives:**

Galactose-deficient IgA1 (Gd-IgA1) is a critical effector molecule in the pathogenesis of IgA nephropathy (IgAN), a leading renal disease without noninvasive assessment options. This updated systematic review aimed to determine the diagnostic and prognostic value of Gd-IgA1 assessment in biological fluids in patients with IgAN.

**Methods:**

PRISMA guidelines were followed in this review. We searched PubMed, Embase, Cochrane Library, China National Knowledge Infrastructure, China Biology Medicine disc, VIP Information/China Science and Technology Journal Database, and WANFANG for studies published between database inception and January 31, 2023. Eligible studies that evaluated aberrant IgA1 glycosylation in IgAN patients relative to controls were identified, and random effects meta-analyses were used to compare Gd-IgA1 levels in different groups. The quality of the evidence was assessed using the Newcastle-Ottawa Scale. This study was registered on PROSPERO (CRD42022375246).

**Findings:**

Of the 2727 records identified, 50 were eligible and had available data. The mean Newcastle-Ottawa Scale score was 7.1 (range, 6–8). Data synthesis suggested that IgAN patients had higher levels of blood and/or urine Gd-IgA1 compared with healthy controls (standard mean difference [SMD]=1.43, 95% confidence interval [CI]=1.19−1.68, P<0.00001), IgA vasculitis patients (SMD=0.58, 95% CI=0.22−0.94, P=0.002), and other kidney disease patients (SMD=1.06, 95% CI=0.79−1.33, P<0.00001). Moreover, patients with IgAN had similar levels of serum Gd-IgA1 compared to first-degree relatives (SMD=0.38, 95% CI= -0.04−0.81, P=0.08) and IgA vasculitis with nephritis patients (SMD=0.12, 95% CI= -0.04−0.29, P=0.14). In addition, ten studies demonstrated significant differences in serum Gd-IgA1 levels in patients with mild and severe IgAN (SMD= -0.37, 95% CI= -0.64−-0.09, P=0.009).

**Conclusions:**

High serum and urine Gd-IgA1 levels suggest a diagnosis of IgAN and a poor prognosis for patients with this immunological disorder. Future studies should use more reliable and reproducible methods to determine Gd-IgA1 levels.

**Systematic review registration:**

https://www.crd.york.ac.uk/prospero/display_record.php?ID=CRD42022375246, identifier CRD42022375246.

## 1 Introduction

Immunoglobulin A nephropathy (IgAN) is the most common form of glomerulonephritis worldwide and remains a leading cause of chronic kidney disease and kidney failure. Approximately 25–30% of patients with IgAN develop end-stage kidney disease 20–25 years after kidney biopsy (1).

The exact pathogenesis of IgAN is not yet defined (2). In IgAN, mesangial deposits of IgA contain high concentrations of abnormally O-glycosylated IgA1 characterized by under-galactosylation. IgAN is believed to be closely associated with the mucosal immune system. The formation of galactose-deficient IgA1 (Gd-IgA1) following a mucosal antigen challenge is the first stage of the “multiple-hit pathogenesis” of IgAN (3). To date, increased serum levels of Gd-IgA1 have been reported in up to 90% of patients with IgAN from different cohorts around the globe (4).

A kidney biopsy is the gold standard for the diagnosis and assessment of IgAN disease activity, but for noninvasive diagnosis of this disease and to delineate the risk of progression more fully, reliable biomarkers are needed. Serum Gd-IgA1 represents the most widely studied and most promising candidate biomarker for IgAN (5). A systematic review in 2016 suggested that the concentration of Gd-IgA1 in the serum or in supernatant of cultured cells from peripheral blood or tonsils may predict the onset of IgAN, though the Gd-IgA1 level was not significantly associated with disease severity (6). In recent years, a novel lectin-independent method using the antibody KM55 to measure Gd-IgA1 levels has paved the way for more convincing diagnostic and disease activity assessment of IgAN (7). Moreover, urinary excretion of Gd-IgA1 was shown to discriminate patients with IgAN from those with other kidney diseases, and the urine Gd-IgA1 level correlated with proteinuria in patients with IgAN (8, 9).

An ideal biomarker would be measurable in an easily available source (e.g., blood or urine), be sensitive and specific for the condition, allow for early diagnosis, vary in response to treatment, have prognostic value, and be biologically plausible (10). This review and meta-analysis aimed to determine whether serum or urine Gd-IgA1 can serve as a useful biomarker for the diagnosis of and assessment of prognosis in patients with IgAN.

## 2 Methods

This systematic review was performed according to the Cochrane Handbook for Systematic Reviews of Interventions and Preferred Reporting Items for Systematic Reviews and Meta-Analyses (PRISMA) (S1 File).

### 2.1 Eligibility criteria

The eligible studies had to meet all the following criteria: (1) the design was case-control, cohort, or cross-sectional; (2) patients in one group were diagnosed with primary IgAN via a kidney biopsy; (3) patients in the control group were healthy controls from the community, first-degree relatives of patients with IgAN, or patients with diseases other than IgAN; (4) the study analyzed blood or urine samples from participants; and (5) Gd-IgA1 levels were determined by enzyme-linked immunosorbent assay (ELISA). Subgroups were established first based on the sample (blood or urine), then by the method of Gd-IgA1 detection and patient age.

### 2.2 Information sources

The following databases were searched on January 31, 2023, for applicable references: PubMed, Embase, Cochrane Library, China National Knowledge Infrastructure, China Biology Medicine disc, VIP Information/China Science and Technology Journal Database, WANFANG, and Web of Science. No restrictions were imposed on the publication period. We limited the search to papers published in English or Chinese; unpublished studies were not sought.

### 2.3 Search strategy

The search terms for IgAN included Glomerulonephritides, IGA and Berger’s Disease and Bergers Disease and IGA Glomerulonephritis and Nephropathy, IGA and Iga Nephropathy 1 and Nephropathy 1, Iga and Immunoglobulin A Nephropathy and Nephropathy, Immunoglobulin A and Nephritis, IGA Type and IGA Type Nephritis and Berger Disease and IGA Nephropathy. The search terms for glycosylation included Glycosylations and Protein Glycosylation and Glycosylation, Protein and Glycosylations, Protein and Protein Glycosylations. The search terms for Gd-IgA1 included galactose-deficient IgA1 and Gd-IgA1. The full search strategy for PubMed (https://pubmed.ncbi.nlm.nih.gov/) is presented in **Supplemental Table 1**. The search strategy for other databases was consistent with that for PubMed.

### 2.4 Data extraction

Two researchers replicated the reference research at different times using the descriptors initially defined. Subsequently, two researchers screened all titles and abstracts separately. Only titles and abstracts related to Gd-IgA1 levels measured in patients with IgAN and controls were retained. Subsequently, the researchers compared the references resulting from their separate screening processes. In case of disagreement regarding whether to include a study, a third researcher was consulted to resolve the issue.

Subsequently, the final references obtained in the previous step were read completely. This complete reading extracted the epidemiological, clinical, and laboratory descriptions of patients with IgAN. Information about the publications was checked, including authorship, year of publication, place of study (city or country), and study design. Epidemiological information, including the number of study participants, sex, and age, was obtained.

The following clinical and laboratory characteristics were investigated: serum Gd-IgA1 levels, urinary excretion of Gd-IgA1, detection index of Gd-IgA1, histopathological grading, and other indicators reflective of disease severity.

### 2.5 Study risk of bias assessment

The Newcastle-Ottawa Scale was used to assess the quality of the included studies by judging them using three broad perspectives: the selection of study groups, the comparability of study groups, and the measurement of exposure in study groups.

### 2.6 Data synthesis and statistical analysis

Review Manager 5.4 was applied for the meta-analysis of the extracted data. To reduce methodological heterogeneity, data that could be converted to consistent units, such as from ng/mL to mg/L, was so converted. To combine the effect size when using different scales in the same outcome area, the level of the continuous variable Gd-IgA1 reported in the original literature was transformed into the standard mean difference (SMD) and its 95% confidence interval (CI). If the pooled SMD was > 0 and the 95% CI did not overlap with zero, then P<0.05 and the difference was considered statistically significant. Chi-square and Cochran’s Q tests were used to explore the heterogeneity caused by various factors. The inconsistency index (I-squared) was computed to quantify heterogeneity. I^2^ less than 50% indicated low heterogeneity, I^2^ between 50% and 75% indicated moderate heterogeneity, and I^2^ greater than 75% indicated high heterogeneity. A random effects model was used to consolidate the index. For studies with high heterogeneity, a sensitivity analysis was used to explore its sources, and a targeted subgroup analysis was performed. Finally, reporting bias was assessed by visually examining the funnel plot.

## 3 Results

### 3.1 Study selection and characteristics

**Figure 1:**
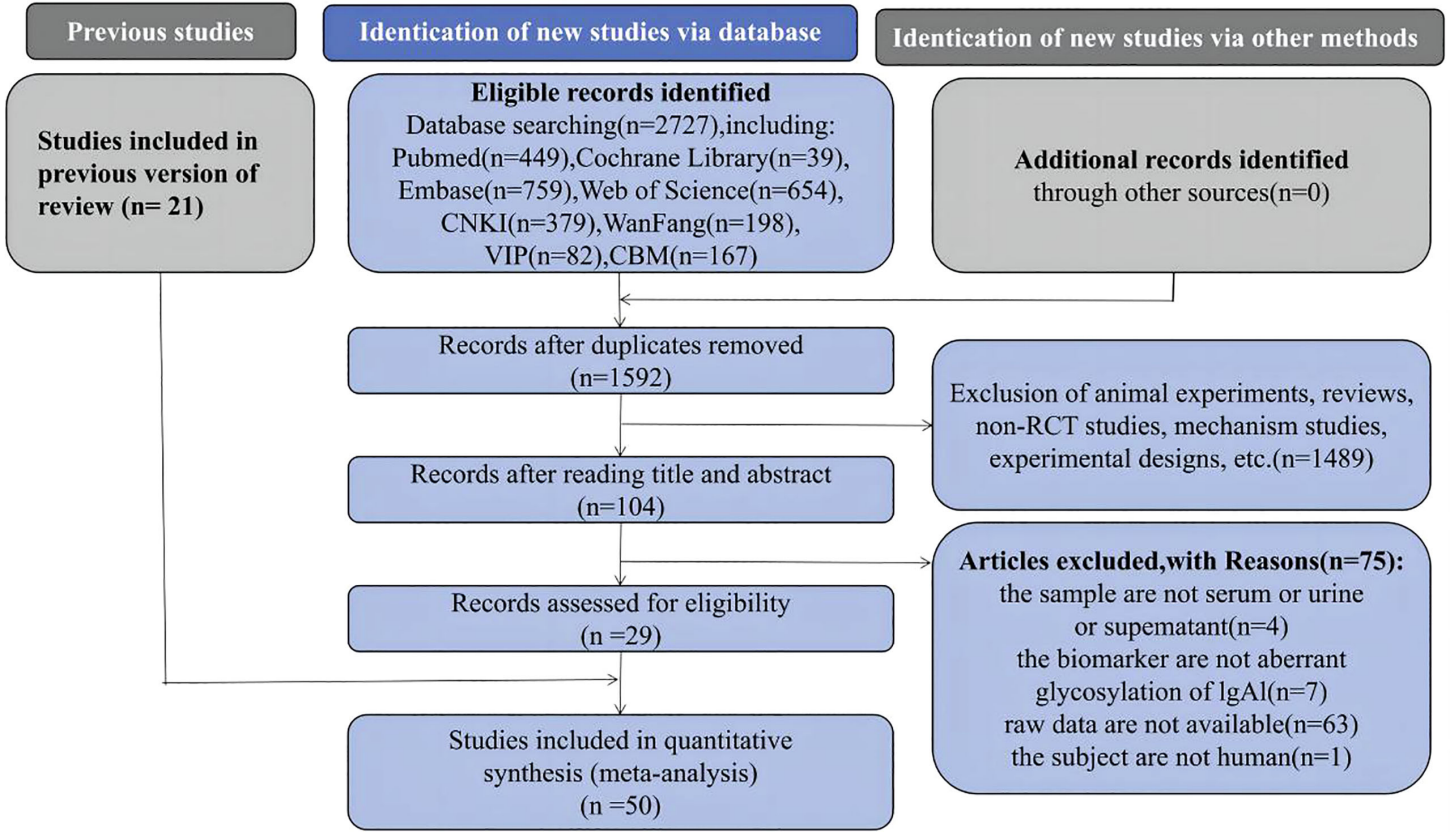
Preferred Reporting Items for Systematic Reviews and Meta-analyses flow diagram of study selection.

In this study, 2727 papers were retrieved. Papers that met the inclusion and exclusion criteria were evaluated and screened via deduplication and reading of abstracts and full texts. Twenty-nine studies were identified for inclusion; adding them to the 21 papers included in the previous systematic review brought the total for this systematic review and meta-analysis to 50 papers (11–51) (**Figure 1**).

Thirty-two of the 50 papers were written in English, 18 were written in Chinese, and all were case-control studies. The 50 papers analyzed included 5263 participants (375 children). Gd-IgA1 from serum, plasma, or urine was detected using either a lectin-dependent assay (e.g., biotinylated N-acetylgalactosamine-specific lectin from Helix aspersa [HAA], biotinylated Vicia villosa lectin [VVL], or biotinylated helix pomatia [HPA]) or an assay using Gd-IgA1-specific antibody (e.g., KM55 or 35A12). The characteristics of the included studies are summarized in **Supplemental Table 2**.

### 3.2 Risk of bias

The Newcastle-Ottawa Scale was used to evaluate the quality of the included studies. The maximum score was 9, and the average score of the 50 studies was 7.1 (range 6–8). The presence of eight low-quality papers with scores of 6 and 42 high-quality papers with scores of 7–8 indicated that the overall quality of the papers was high and the risk of bias was low (**Supplemental Table 3**).

### 3.3 IgAN versus healthy controls

A total of 43 studies with 3671 samples compared patients with IgAN to healthy controls. The results showed that patients with IgAN had higher levels of Gd-IgA1 in blood and urine compared to healthy controls (P<0.00001, Heterogeneity: I^2^ = 89% >75%) (**Supplemental Figure 1**). Subgroups were established according to the different sample types: urine and blood.

Three studies involving 389 samples were included in the urine sample subgroup. In accordance with the above results, the levels of urine Gd-IgA1 in IgAN patients were significantly higher than those in healthy controls (P<0.00001, Heterogeneity: I^2 ^= 0%) (**Supplemental Figure 2** -1.1.1). Similarly, in the blood subgroup, the levels of Gd-IgA1 in patients with IgAN were significantly higher than those in healthy controls (P<0.00001, Heterogeneity: I^2 ^= 90% >75%) (**Supplemental Figure 2** -1.1.2). Given the high heterogeneity, additional subgroups were established.

**Figure 2:**
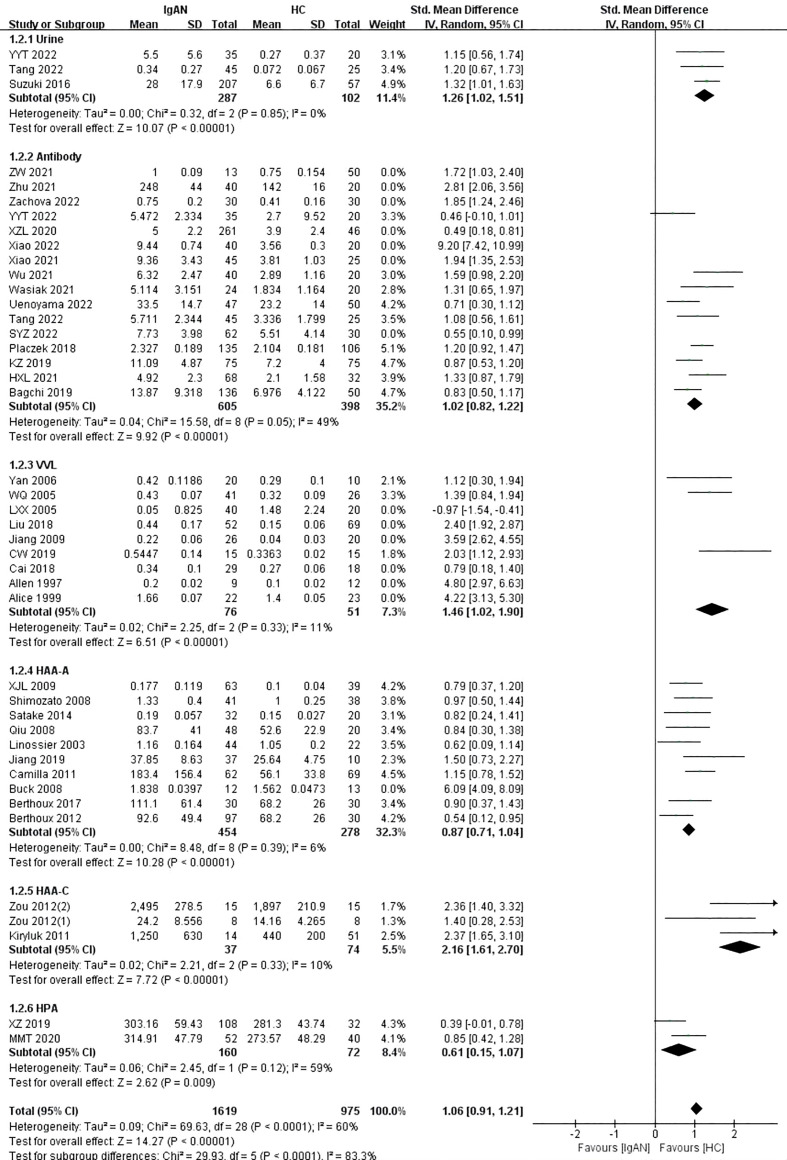
The forest plot of the comparation between IgAN group and HC group.

The blood sample subgroup was divided into four additional subgroups: antibody, HAA, VVL, and HPA. Fifteen studies used the Gd-IgA1 specific antibody KM55, and one study used the 35A12 antibody. The detection of Gd-IgA1 levels by antibody was similar to the above results (P<0.00001, Heterogeneity: I^2 ^= 90%) (**Supplemental Figure 3** -1.2.2). Given that I^2^ was above 75%, a sensitivity analysis was performed to explore the source of the heterogeneity. After excluding SYZ 2022, Xiao 2021, Xiao 2022, XZL 2020, Zachova 2022, Zhu 2021, and ZW 2021, I^2^ was less than 50% and the level of Gd-IgA1 in patients with IgAN was significantly higher than that in healthy controls (P<0.00001, Heterogeneity: I^2 ^= 49%) (**Figure 2** -1.2.2).

In the VVL lectin subgroup, there were nine studies on children and adults in which 467 participants were included. Patients with IgAN had significantly higher levels of Gd-IgA1 compared with healthy controls (P=0.0001, Heterogeneity: I^2 ^= 95% >75%) (**Supplemental Figure 3** -1.2.3). Given the high heterogeneity of the VVL subgroup, a sensitivity analysis was performed. When the six studies by Alice 1999, Allen 1997, Cai 2018, Jiang 2009, Liu 2018, and LXX 2005 were excluded, I^2 ^= 11% and the result remained unchanged (P<0.00001) (**Figure 2**-1.2.3).

In the HAA lectin subgroup, there were three studies on children (111 participants) and 10 studies on adults (757 participants). Adults with IgAN had significantly higher levels of Gd-IgA1 compared with healthy adults (P<0.00001, Heterogeneity: I^2 ^= 74% >50%) (**Supplemental Figure 3**-1.2.4). Heterogeneity decreased, and the result was maintained after excluding the study by Buck 2008 (P<0.00001, Heterogeneity: I^2 ^= 6%) (**Figure 2**-1.2.4). Children with IgAN also had significantly higher levels of Gd-IgA1 compared with healthy children (P<0.00001, Heterogeneity: I^2 ^= 10%) (**Figure 2**-1.2.5).

Two studies involving 232 participants were included in the HPA lectin subgroup. The levels of Gd-IgA1 in patients with IgAN were higher than those in healthy controls (P=0.009, Heterogeneity: I^2 ^= 59%) (**Figure 2**-1.2.6).

### 3.4 IgAN versus first-degree relatives

**Figure 3:**
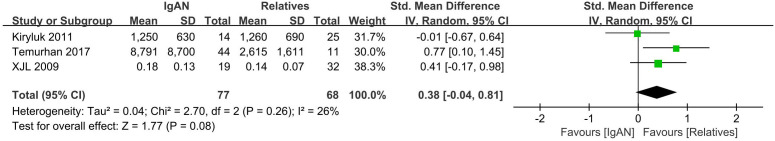
The forest plot of IgAN group and first-degree relatives group.

Three studies with a total of 145 participants were included in this portion of the meta-analysis. There were no differences in serum Gd-IgA1 levels between IgAN patients and their first-degree relatives (P=0.08, Heterogeneity: I^2 ^= 26%) (**Figure 3**).

### 3.5 IgAN versus immunoglobulin A vasculitis versus immunoglobulin A vasculitis with nephritis

**Figure 4:**
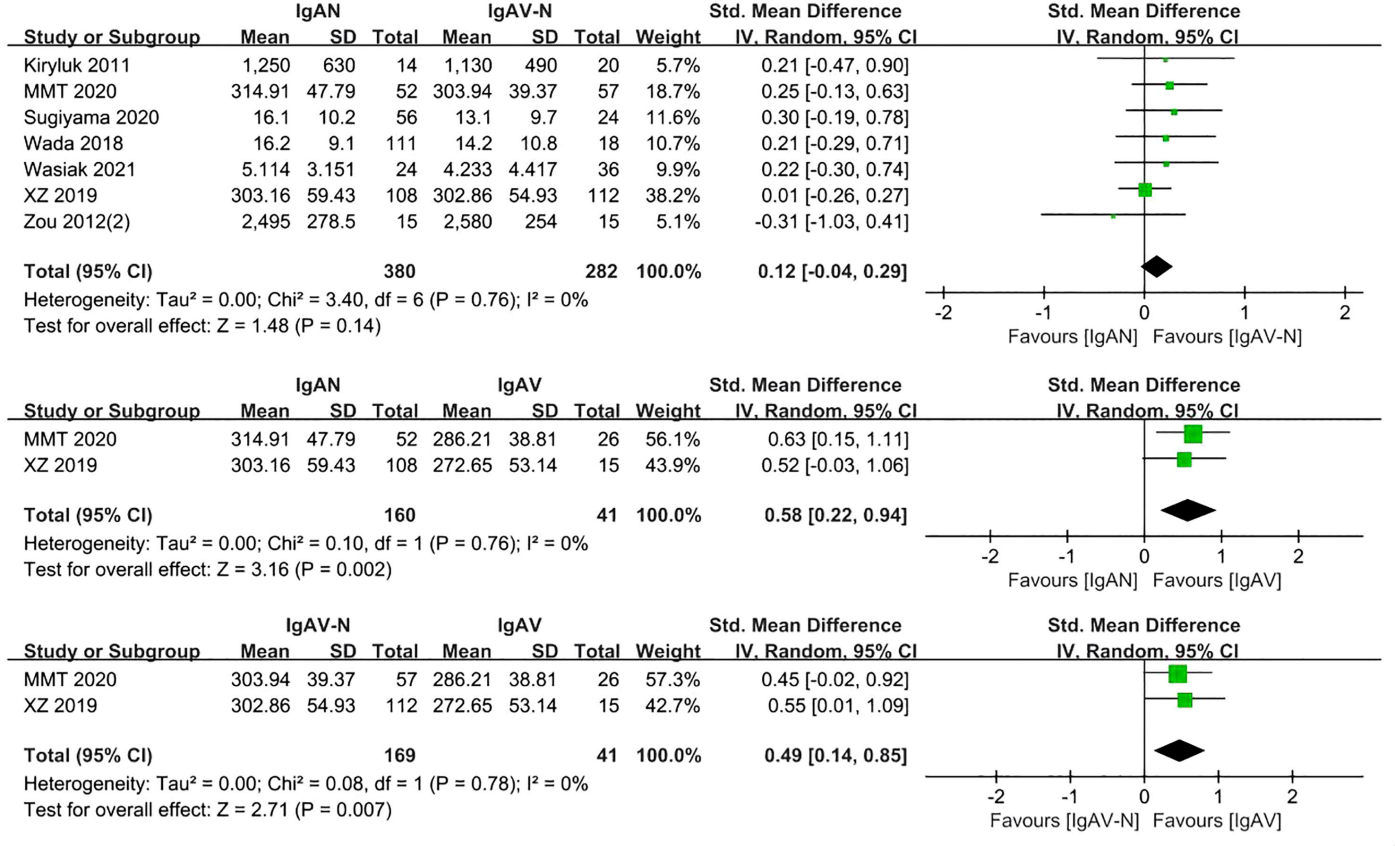
The forest plot of the comparation among IgAN group, IgAV-N group and IgAV group.

Immunoglobulin A vasculitis (IgAV) was formerly known as Henoch-Schönlein purpura. Once IgAV that has affected the small blood vessels of the kidney, it is known as IgAV with nephritis (IgAV-N). Seven included studies involving 662 participants compared serum Gd-IgA1 levels of patients with IgAN and IgAV-N. There were no significant differences in Gd-IgA1 levels in this comparison (P=0.14, Heterogeneity: I^2 ^= 0%) (**Figure 4**).

In the two studies that included patients with IgAN, IgAV, and IgAV-N (370 participants), the levels of serum Gd-IgA1 in IgAN and IgAV-N patients were higher than those in IgAV patients (P=0.002, Heterogeneity: I^2 ^= 0%; P=0.007, Heterogeneity: I^2 ^= 0%) (**Figure 4**).

**Figure 5:**
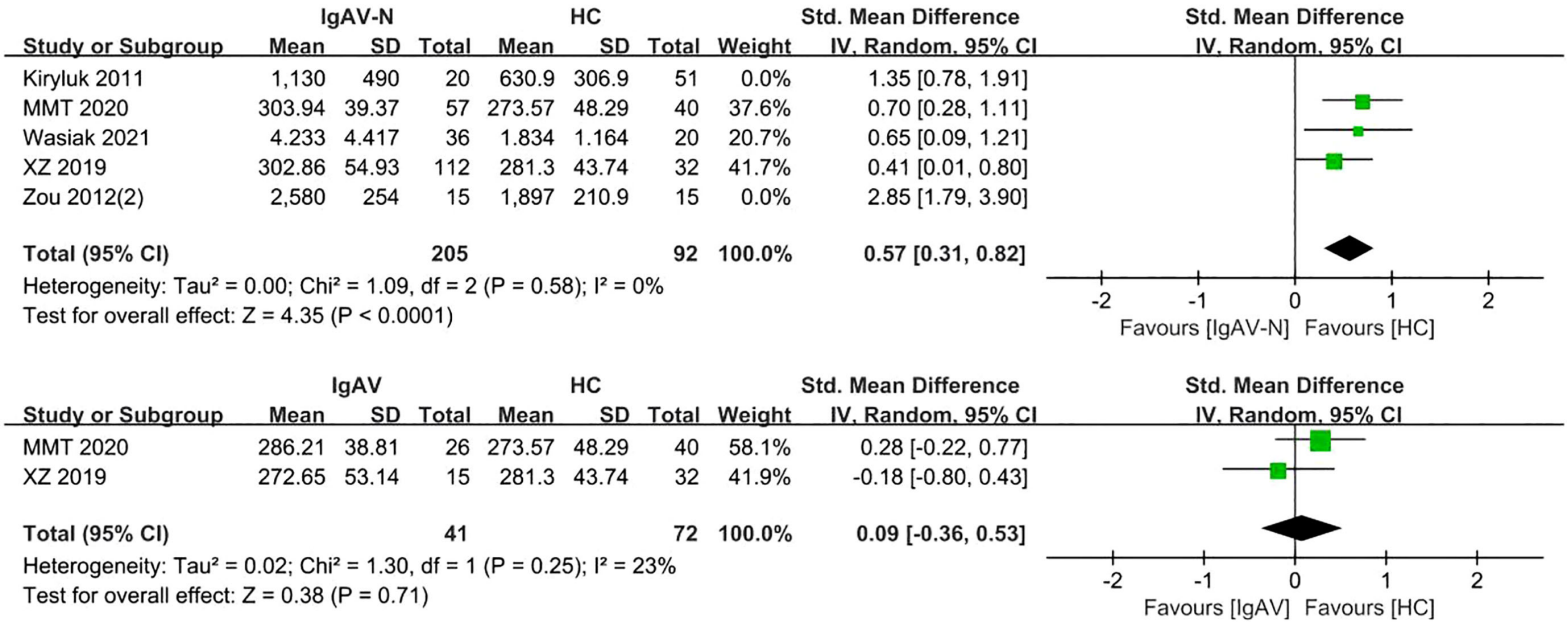
The forest plot of the comparation among IgAV-N group, IgAV group and HC group.

Analysis of five studies comprising 398 samples demonstrated levels of serum Gd-IgA1 in patients with IgAV-N that were significantly greater than those in healthy controls (P= 0.0003, Heterogeneity: I^2 ^= 82% >75%) (**Supplemental Figure 4**); the heterogeneity decreased while the result was maintained after excluding Kiryluk 2011 and Zou 2012(2) (P<0.0001, Heterogeneity: I^2 ^= 0%) (**Figure 5**).

Two studies involving 113 adults and children were included. Patients with IgAV had similar levels of serum Gd-IgA1 compared to healthy controls (P=0.71, Heterogeneity: I^2 ^= 23%) (**Figure 5**).

### 3.6 IgAN versus other kidney diseases

A total of 24 studies involving 2488 participants were included. The forest plot showed that the IgAN patients had obviously higher levels of Gd-IgA1 compared to other kidney disease patients (minimal change nephrotic syndrome, primary glomerular disease, minimal change nephrotic, lupus nephritis, mesangial proliferative glomerulonephritis, membranous nephropathy, focal segmental glomerulosclerosis, membranoproliferative glomerulonephritis, and mesangiocapillary glomerulonephritis) (P<0.00001, Heterogeneity: I^2 ^= 88% >75%) (**Supplemental Figure 5**). Subgroups were established according to the different detection methods for Gd-IgA1: antibody, VVL, and HAA.

**Figure 6:**
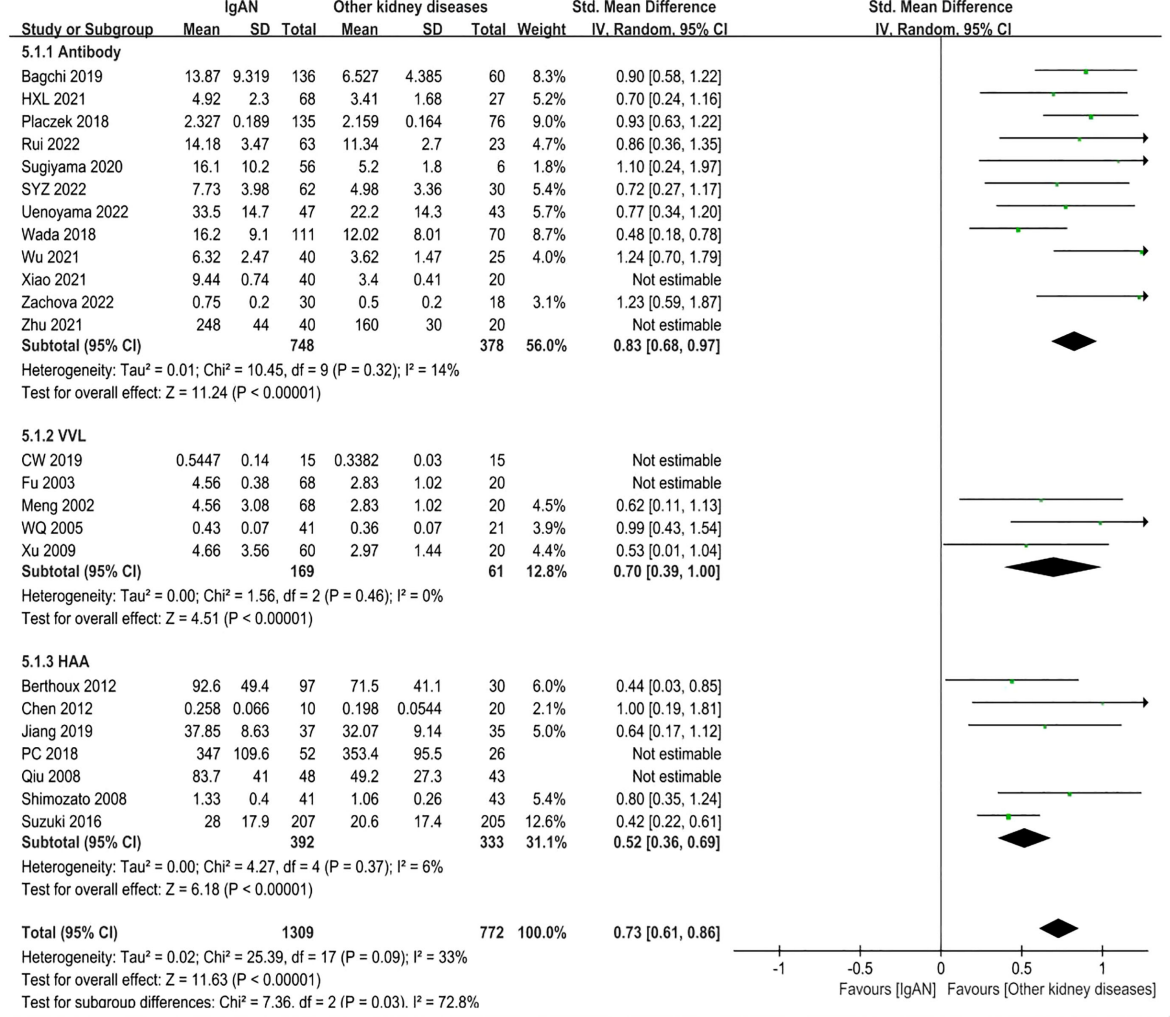
The forest plot of IgAN group and other kidney diseases group.

In the antibody subgroup, 1246 participants were included in 12 studies. In similar results as above, IgAN patients had higher Gd-IgA1 levels compared with other kidney diseases patients (P<0.00001; Heterogeneity: I^2 ^= 90% >75%) (**Supplemental Figure 6** -5.1.1). After eliminating Xiao 2021 and Zhu 2021 by sensitivity analysis, the heterogeneity decreased (I^2 ^= 14%) and the result remained unchanged (P<0.00001) (**Figure 6** -5.1.1).

In VVL lectin subgroup, a total of 5 studies involving 348 participants were included. In accordance with the above result, the level of Gd-IgA1 in IgAN patients was higher than that in patients with other kidney diseases (P=0.002, Heterogeneity: I^2 ^= 90% >75%) (**Supplemental Figure 6** -5.1.2). The heterogeneity decreased (I^2 ^= 0%) when two trials (CW 2019, Fu 2003) were excluded, and the result remained the same (P<0.00001) (**Figure 6**-5.1.2).

In HAA lectin subgroup, there were 7 studies in which 894 participants were included. Patients with IgAN had higher levels of Gd-IgA1 compared to other kidney diseases (P<0.00001, Heterogeneity: I^2 ^= 58% >50%) (**Supplemental Figure 6** -5.1.3). After excluding PC 2018 and Qiu 2008, I^2 ^= 6% and the result remained the same (P<0.00001) (**Figure 6**-5.1.3).

### 3.7 Other kidney diseases versus healthy controls

Sixteen studies involving 1265 adult participants were included. There were differences in Gd-IgA1 levels between patients with other kidney diseases and healthy controls (P=0.008, Heterogeneity: I^2 ^= 64% >50%) (**Supplemental Figure 7**). Subgroup analysis was performed in accordance with the different detection methods for Gd-IgA1.

**Figure 7:**
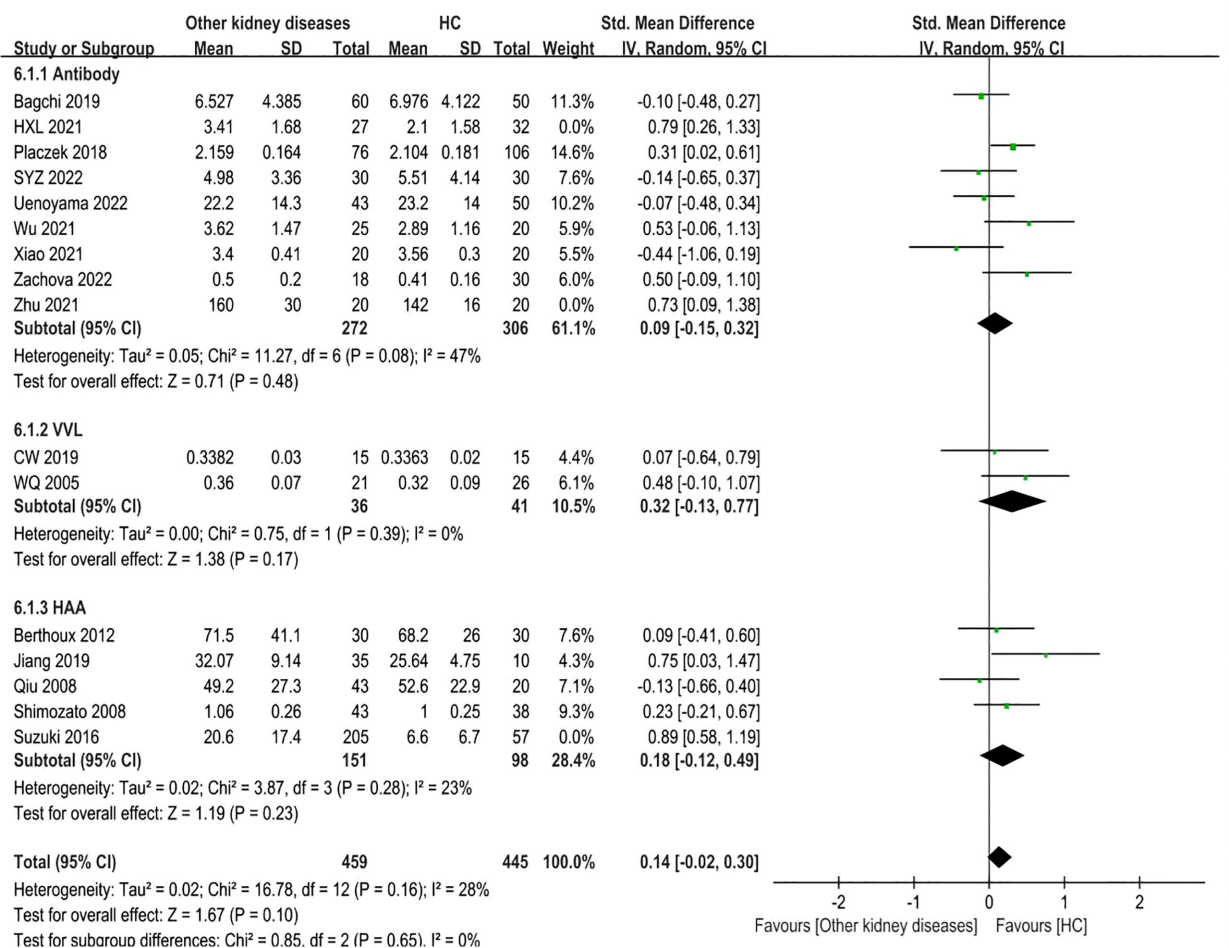
The forest plot of other kidney diseases group and HC group.

In the antibody subgroup, nine studies with 677 participants were included. In contrast to the above result, there were no differences in Gd-IgA1 levels between other kidney diseases patients and healthy controls (P=0.10, Heterogeneity: I^2 ^= 60% >50%) (**Supplemental Figure 8**-6.1.1). Sensitivity analysis resulted in I^2 ^= 47% after two trials (HXL 2021, Zhu 2021) were excluded, while the results remained the same (P=0.48) (**Figure 7**-6.1.1).

In the VVL lectin subgroup, two studies involving 77 participants were included. There were also no statistical differences in Gd-IgA1 levels between the two groups (P=0.17, Heterogeneity: I^2 ^= 0%) (**Figure 7**-6.1.2).

Five studies comprising 511 samples were included in the HAA lectin subgroup. Other kidney disease patients had similar levels of Gd-IgA1 compared to the healthy controls (P=0.08, Heterogeneity: I^2 ^= 75% >50%) (**Supplemental Figure 8**-6.1.3). After excluding Suzuki 2016 by sensitivity analysis, the heterogeneity decreased (I^2 ^= 23%) (**Figure 7**-6.1.3) and the result remained (P=0.23). Therefore, the above results showed no differences in the Gd-IgA1 levels between patients with other kidney diseases and healthy controls.

### 3.8 IgAN and severity

Ten studies with 465 participants were included to compare the Gd-IgA1 level in cases of mild versus severe IgAN. The level of Gd-IgA1 in mild IgAN was lower than that in severe IgAN (P=0.009, Heterogeneity: I^2 ^= 47%) (**Supplemental Figure 9**). Six comparisons were based on histopathological grading: mild mesangial proliferative IgAN and focal proliferative sclerosing IgAN; I + II vs IV + V; I– III vs IV– V; I vs III (1982 WHO classification). The other four comparisons were based on a combination of several risk factors that could reflect the severity of IgAN to some extent. One was based on histopathological grading and estimated glomerular filtration rate (eGFR): I-III with eGFR ≥ 60 ml/min versus III with eGFR < 60 ml/min. One was based on absolute renal risk (ARR) for ultimate dialysis or death: 1 versus 3. The ARR was estimated based on proteinuria, blood pressure, and light microscopic features on renal biopsy. One was based on the clinical grade according to the values of proteinuria and eGFR, which were determined using the criteria of the Japanese Society of Nephrology (JSN): I versus III. The last was based on risk stratification of progression to end-stage kidney disease: low-risk versus high-risk. Risk stratification was determined based on a combination of clinical and histological grades according to the JSN. Subgroups were established according to the different classification methods used to classify the grades of IgAN severity: histopathological grading and other risk factor subgroups.

**Figure 8:**
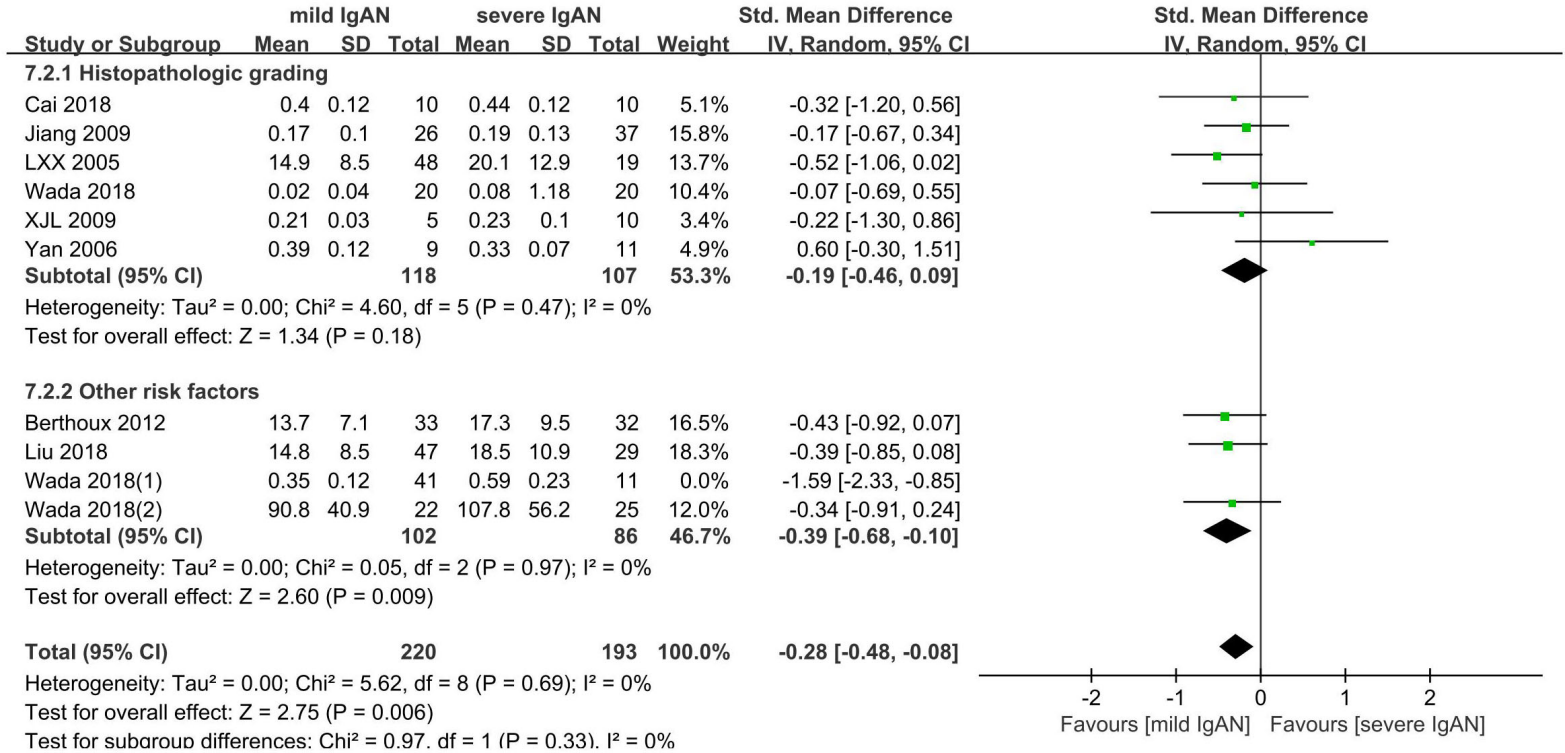
The forest plot of comparison among variable grades of IgAN severity.

In the histopathological grading subgroup, six studies involving 225 participants were included. There were no significant differences in serum Gd-IgA1 levels between patients with severe IgAN and mild IgAN (P=0.18; Heterogeneity: I^2 ^= 0%) (**Figure 8**-7.2.1).

The other risk factor subgroup included four comparisons involving 240 participants. The level of Gd-IgA1 in mild IgAN was lower than that in severe IgAN (P=0.01, Heterogeneity: I^2 ^= 66% >50%) (**Supplemental Figure 10**). I^2 ^= 0% after one trial (Wada 2018(1)) was excluded, and the results remained the same (P=0.009) (**Figure 8**-7.2.2).

## 4 Discussion

### 4.1 Summary of main findings

In the current meta-analysis, patients with IgAN had significantly higher blood and urine levels of Gd-IgA1 than healthy controls, which is consistent with the results of a systematic review conducted in 2016 (6). This indicates that blood or urine Gd-IgA1 levels have diagnostic value for IgAN. There is good evidence that the clinical presentation, disease progression, and long-term outcome of IgAN differ across ethnic populations around the world (1). For example, more severe clinical presentation and higher risk of disease progression have been reported in Asians than Europeans. Moreover, active lesions, such as endocapillary hypercellularity and crescents, are more commonly reported in Asians than Europeans (4). Interestingly, Gd-IgA1 levels in Chinese IgAN patients are lower than those found in European healthy controls (52), suggesting that there are racial differences in Gd-IgA1 levels, that race plays an important role in the diagnostic role of Gd-IgA1, and that patients with IgAN must be compared with ethnically matched healthy subjects. In addition, many researchers have explored the cutoff values of serum or urine Gd-IgA1 for diagnosing IgAN (53–56). For example, Tang Y et al. found that serum and urine Gd-IgA1 concentration could distinguish patients with IgAN from healthy controls, and the best cutoff values for serum and urine Gd-IgA1 were 2,876.2 ng/mL (sensitivity, 97%; specificity, 70%) and 0.745 ng·l/ml·μmol (sensitivity, 94%; specificity, 95%), respectively, which suggested that urine Gd-IgA1 has a greater diagnostic value for IgAN than serum Gd-IgA1 (53).

However, there was a significant overlap in serum Gd-IgA1 levels between patients with IgAN and healthy controls. Many, but not all, patients with IgAN have elevated serum Gd-IgA1 levels. Yanagawa et al. suggested that the assessment of serum levels of Gd-IgA1 together with serum levels of Gd-IgA1-specific antibodies could improve the specificity of IgAN diagnosis (55). High serum and urine Gd-IgA1 levels are suggestive of IgAN but are not substitutes for kidney biopsy. ELISA Gd-IgA1 evaluation can be routinely in the examination of patients with CKD and proteinuria/hematuria suspected of IgAN, especially those who do not want renal biopsy or have contraindications to renal biopsy, which can guide clinical treatment to some extent. In particular, urine Gd-IgA1 levels may be a biomarker for the early screening of potential IgAN (56).

### 4.2 Findings in the context of other literature

In previous studies, Gd-IgA1 has been detected using ELISA or mass spectrometry. Mass spectrometry is not suitable for clinical applications because of complicated sample preparation (57). Studies have shown that ELISA using the Gd-IgA1-specific monoclonal antibody KM55 can reliably and reproducibly evaluate Gd-IgA1 compared with lectin-dependent methods, and thus could serve as a powerful tool with which to clarify this unpredictable disease (7). However, only a few large-scale studies have used this method to measure Gd-IgA1 levels. We recommend this novel lectin-independent method using KM55 to detect serum and urine levels of Gd-IgA1 in clinical practice in the future.

Interestingly, many unaffected relatives of IgAN patients also had elevated Gd-IgA1 levels, which was consistent with the results of a systematic review in 2016 (6) suggesting that high levels of serum Gd-IgA1 are heritable in IgAN, further suggesting that Gd-IgA1, which is potentially important in the pathogenesis of IgAN, is not sufficient to cause the disease. Over the past 50 years, familial aggregation of IgAN has been reported in different races. Previous genome-wide association studies (GWAS) have identified the major susceptibility loci for IgAN, but the underlying pathogenic genes have not yet been isolated at these loci, and their roles in the development of IgAN are still unknown. Isolation of the underlying pathogenic genes will provide new targets for the prevention and treatment of IgAN. In addition, researchers found that the serum Gd-IgA1 levels in patients with IgAN were markedly higher than those in their spouses, whereas there were no differences between the patients’ spouses and normal controls (58). This indicates that environmental factors may play little role in the production of serum Gd- IgA1.

In our study, no significant differences in serum Gd-IgA1 levels were found between patients with IgAN and IgAV-N, which is consistent with previous studies (6). Serum Gd-IgA1 levels were significantly higher in patients with IgAN and IgAV-N than in patients with IgAV and healthy controls. IgAV, formerly known as Henoch-Schönlein purpura, is a form of vasculitis characterized by IgA deposition within the blood vessels of the affected tissues. Its pathogenesis is complex and has not yet been fully elucidated. IgAV commonly affects the small blood vessels in the kidneys. Kidney involvement in IgAV is histopathologically indistinguishable from that in IgAN. IgAN and IgAV-N are considered related diseases that share similar clinicopathological phenotypes (1). Xu et al. suggested that the formation of Gd-IgA1 and related immune complexes plays a vital role in promoting the occurrence and development of IgAV-N (59). Neufeld et al. found that both IgAV-N and IgAV patients revealed perivascular and skin Gd-IgA1 deposition. Moreover, high Gd-IgA1 levels in patients with IgAV-N suggest a dose-dependent effect of Gd-IgA1 in IgAV (60). Li et al. found that the combined detection of circulating zonulin and Gd-IgA1 may be a non-invasive diagnostic biomarker for IgAV-N and IgAN (61). Therefore, we believe that serum Gd-IgA1 levels may also be a useful tool for screening IgAV-N, as well as IgAN. When compared to the baseline serum Gd-IgA1 level, an elevated serum Gd-IgA1 level might predict kidney damage in patients with IgAV. However, further clinical trials are needed to confirm this hypothesis. In addition, there was no significant difference in plasma Gd-IgA1 levels in IgAV patients compared with healthy subjects in this study, which we believe is a result of the small sample size of IgAV patients included, and the sample size should be expanded in the future to further explore the changes in plasma Gd-IgA1 levels in IgAV patients.

In recent years, more clinical studies have compared the differences in serum or urine Gd-IgA1 levels between IgAN and other kidney diseases such as minimal change disease and membranous nephropathy. In the current study, twenty-four studies indicated that serum and urine Gd-IgA1 levels in IgAN patients were significantly higher than those in patients with other kidney diseases. Furthermore, there were no significant differences in Gd-IgA1 levels between patients with other kidney diseases and healthy controls, suggesting the diagnostic potential of Gd-IgA1 in discriminating patients with IgAN from those with other kidney diseases and healthy controls.

The Oxford pathological score, eGFR, proteinuria, hypertension, and many other risk factors reflect the severity of IgAN to some extent. However, there are no validated prognostic serum or urine biomarkers for IgAN, other than eGFR and proteinuria (1). Some studies have shown that serum Gd-IgA1 levels do not correlate with clinical and histological characteristics, and cannot predict disease progression. For example, Bagchi et al. and Tang et al. found that serum Gd-IgA1 levels were not correlated with baseline eGFR, urine protein creatinine ratio, proteinuria, and M, E, S, T, and C scores on kidney biopsy, and did not observe a statistically significant correlation between serum Gd-IgA1 levels and renal survival (62, 63). In contrast, many studies have found significant correlations between Gd-IgA1 levels and clinical and pathological features and suggested that Gd-IgA1 could help identify patients with IgAN who will have a poor prognosis and require intensive treatment. Tang et al. observed significant correlations between urine Gd-IgA1 levels and eGFR, proteinuria, and MEST-C scores (53). Maixnerova et al. suggested that a higher serum Gd-IgA1 level predicts faster eGFR decline and poor renal survival (64). Wada et al. also found that serum Gd-IgA1 levels were significantly higher in IgAN patients with glomerular sclerosis and tubulointerstitial lesions, and that mesangial Gd-IgA1 intensity was negatively correlated with eGFR in IgAN (65). In addition, the risk of chronic kidney disease (CKD) progression events was greater with higher plasma Gd-IgA1 levels but reached a plateau when Gd-IgA1 >325 U/ml, whereas the risk of CKD progression events monotonically increased with a higher Gd-IgA1/C3 ratio (66).

In this study, significant differences were found between serum Gd-IgA1 levels in patients with mild and severe IgAN, contrary to the results of a systematic review in 2016 (6). The severity of IgAN was classified based on the clinical manifestations and pathology. Among these, six comparisons were simply based on histopathological grading, whereas the other four were based on two or more traditional risk factors, including histopathological grading, proteinuria, eGFR, and hypertension. Consequently, we established subgroups on this basis, and found that there were no significant differences in serum Gd-IgA1 levels between patients with mild and severe IgAN classified by histopathological grading only, while serum Gd-IgA1 levels differed significantly between patients with mild and severe IgAN classified by two or more risk factors. Therefore, we considered that classifying IgAN severity by histopathological grading alone was inadequate. In addition, we suspect that serum Gd-IgA1 may be a sensitive marker for determining disease severity and prognosis. The clinical and histologic data elements at biopsy are included in the International IgAN Prediction Tool, users can use it to determine a 50% decline in eGFR or kidney failure at selected time intervals (1). We suggest that the International IgAN Prediction Tool should be actively used in future studies to assess the disease severity and prognosis of IgAN patients, and then explore the correlation with Gd-IgA1 levels.

Various targeted therapies have been developed for IgAN, and the selection of targeted therapies requires biomarkers that can accurately predict their efficacy. Whether Gd-IgA1 levels reflect clinical efficacy, such as remission and recurrence, in patients with IgAN who receive therapies is of great concern to clinicians. Atacicept, a blocker of BLyS and APRIL, has the potential to improve proteinuria and renal function and has an acceptable safety profile in patients with IgAN. Importantly, atacicept treatment led to dose-dependent reductions in serum Gd-IgA1, and the reduced Gd-IgA1 level was significantly correlated with improvements in proteinuria (67). Additionally, some studies have shown that the recurrence of IgAN in allograft kidneys is associated with high serum Gd-IgA1 levels (68–70). Moreover, serum Gd-IgA1 level may be a potential biomarker for the diagnosis and activity assessment of recurrent IgAN after kidney transplantation (71–73). However, Jäger et al. found that the serum concentration of Gd-IgA1 within the first year after transplantation had no significant effect on the recurrence of IgAN (74). Therefore, the predictive value of serum Gd-IgA1 level for IgAN recurrence could not be confirmed. Resolving the question of whether serum Gd-IgA1 levels reflect clinical efficacy in patients with IgAN who receive therapies requires larger trials in the future. Elucidating this relationship will help improve therapeutic modalities for IgAN.

### 4.3 Limitations

**Figure 9:**
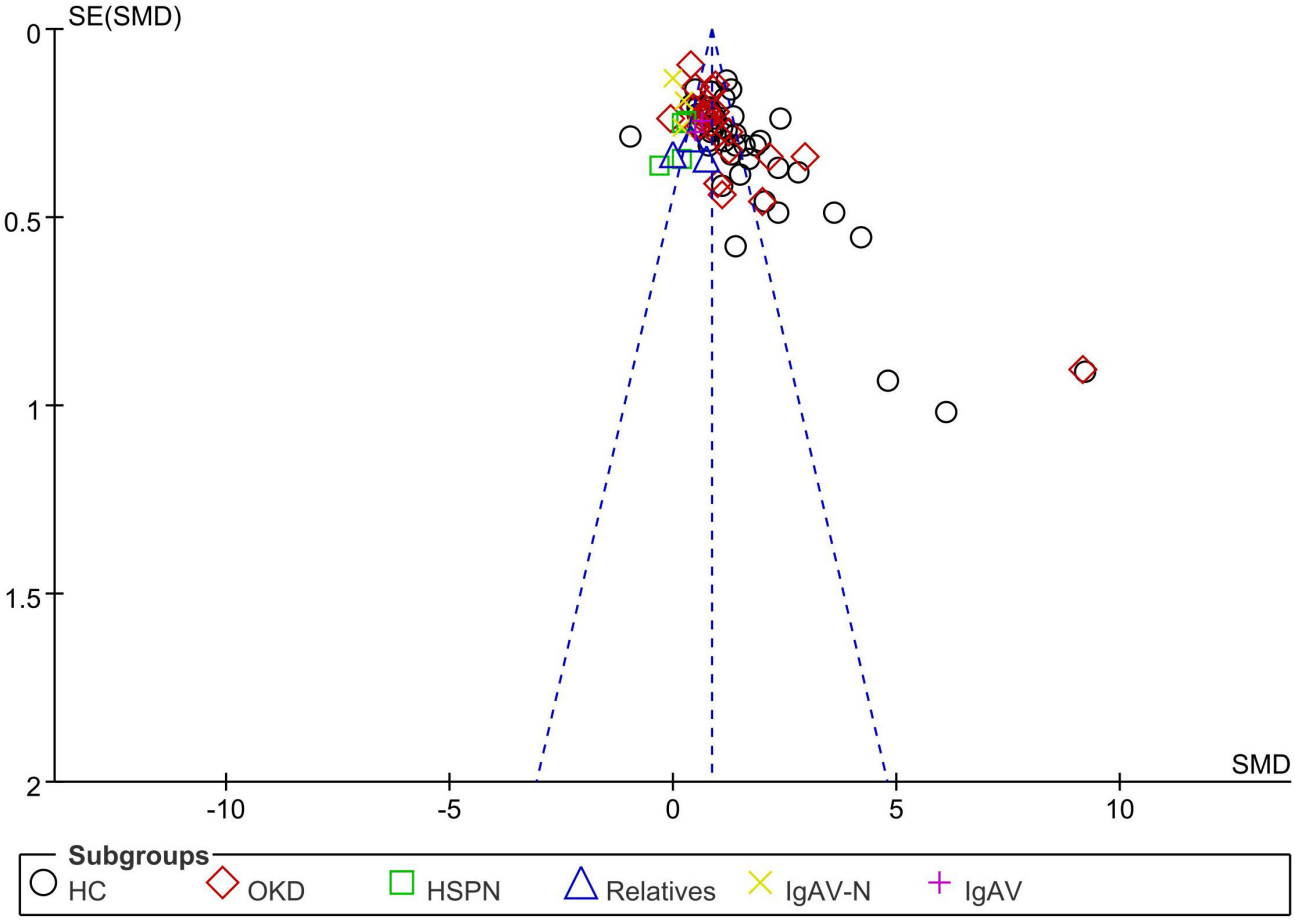
The funnel plot of the meta-analysis.

This study has several limitations. First, the retrieval language was confined to English or Chinese; therefore, publications in other languages may have been missed. Second, the included studies were published, which could have introduced a reporting bias. Visual inspection of the funnel plot showed that most points were concentrated in the upper part of the funnel plot, indicating less evidence of publication bias (**Figure 9**). Finally, our primary problem was that the results showed a high degree of statistical heterogeneity. Therefore, the results should be interpreted with caution.

### 4.4 Conclusions

High serum and urine Gd-IgA1 levels suggest a diagnosis of and poor prognosis for patients with IgAN. Therefore, the utility of serum and urine Gd-IgA1 levels should be assessed in larger patient cohorts from geographically distinct areas. Future studies should use more reliable and reproducible methods to determine Gd-IgA1 levels.

## Author contributions

QZ and W-RW contributed equally to this work. All authors contributed to the article and approved the submitted version.
