# Supplementary material for: Diagnostic and prognostic value of galactose-deficient IgA1 in patients with IgA nephropathy: an updated systematic review with meta-analysis
Source: Front Immunol. 2023 Aug 21;14:1209394. doi: 10.3389/fimmu.2023.1209394 (PMC10475574; doi:10.3389/fimmu.2023.1209394)
Supplement: Supplementary file 1 [file DataSheet_1.docx]

**Supplementary Material**

Supplemental Table 1 Detailed search strategy of PubMed

| Search | Query | Items found |
| --- | --- | --- |
| #1 | Search "Glomerulonephritis, IGA"[Mesh] | 6941 |
| #2 | Search ((((((((((((Glomerulonephritides, IGA[Title/Abstract]) OR (Berger's Disease[Title/Abstract])) OR (Bergers Disease[Title/Abstract])) OR (IGA Glomerulonephritis[Title/Abstract])) OR (Nephropathy, IGA[Title/Abstract])) OR (Iga Nephropathy 1[Title/Abstract])) OR (Nephropathy 1, Iga[Title/Abstract])) OR (Immunoglobulin A Nephropathy[Title/Abstract])) OR (Nephropathy, Immunoglobulin A[Title/Abstract])) OR (Nephritis, IGA Type[Title/Abstract])) OR (IGA Type Nephritis[Title/Abstract])) OR (Berger Disease[Title/Abstract])) OR (IGA Nephropathy[Title/Abstract]) | 8683 |
| #3 | Add search (#1 OR #2) | 9763 |
| #4 | Search "Glycosylation"[Mesh] | 35888 |
| #5 | Search ((((Glycosylations[Title/Abstract]) OR (Protein Glycosylation[Title/Abstract])) OR (Glycosylation, Protein[Title/Abstract])) OR (Glycosylations, Protein[Title/Abstract])) OR (Protein Glycosylations[Title/Abstract]) | 40459 |
| #6 | Add search (#4 OR #5) | 59438 |
| #7 | Search "galactosyl-deficient IgA1" [Supplementary Concept] | 1 |
| #8 | Search (galactose-deficient IgA1[Title/Abstract]) OR (Gd-IgA1[Title/Abstract]) | 252 |
| #9 | Add search (#7 OR #8) | 252 |
| #10 | Add search (#6 OR #9) | 59638 |
| #11 | Add search (#3 AND #10) | 449 |

Supplemental Table 2 Characteristics of studies included in the systematic review and meta-analysis.

| **Serial number mber** | **Publish year** | **First author** | **Country** | **Study Population** | **Mean age(Years)** | **Male (%)** | **Sample** | **Detection Index** | **Unit** |
| --- | --- | --- | --- | --- | --- | --- | --- | --- | --- |
|  |  |  |  |  |  |  |  |  |  |
| **1[11]** | **2012** | **Chen W** | **CN** | **10H-IgAN;10MCNS-IgA;10MCNS** | **28.2;31.5;45.9** | **5(50%);6(66.7%);7(70%)** | **serum** | **HAA** | **NA** |
| **2[12]** | **2022** | **Tang YY** | **CN** | **45IgAN;25HC** | **45.3;42.7** | **25(55.6%);12(48%)** | **serum** | **KM55** | **μg / L** |
|  |  |  |  |  |  |  | **urine** | **KM55** | **μg / L** |
| **3[13]** | **2006** | **Yan Y** | **CN** | **10mmpIgAN;10fpsIgAN;10HC** | **NA** | **NA** | **serum** | **VVL** | **NA** |
| **4[14]** | **2008** | **Qiu Q** | **CN** | **48IgAN;43PGD(22MesPGN;7MCD;5MCG;4MN;2FSGS;4SG);20HC** | **32;34** | **27(56.25%);21(48.83%)** | **serum** | **HAA** | **U** |
| **5[15]** | **2022** | **Y.-Y.T** | **CN** | **35IgAN;20HC** | **40.6;39.1** | **18(51.4%);11(55%)** | **serum** | **KM55** | **ng / mL** |
|  |  |  |  |  |  |  | **urine** | **KM55** | **ng·l/ml·μmol** |
| **6[16]** | **2020** | **X.-Z.L** | **CN** | **261IgAN;46HC** | **37.7;38** | **135(51.7%);22(47.8%)** | **plasma** | **KM55** | **μg/mL** |
| **7[17]** | **2012** | **Zou MS** | **CN** | **8IgAN;8HC** | **9.2;9.5** | **5(62.5%);5(62.5%)** | **serum** | **HAA** | **x10^5^U/L** |
| **8[18]** | **2022** | **Xiao Y** | **CN** | **45IgAN;25HC** | **45.3;42.7** | **25(55.6%);12(48%)** | **serum** | **KM55** | **mg/L** |
| **9[19]** | **2002** | **Meng LQ** | **CN** | **68IgAN;20MCD** | **30.5;23.1** | **40(58.8%);11(55%)** | **serum** | **VVL** | **AU/mg** |
| **10[20]** | **2003** | **Fu SX** | **CN** | **68IgAN;20MCD** | **30;23** | **39(57.4%);11(55%)** | **serum** | **VVL** | **AU/mg** |
| **11[21]** | **2012** | **Zou MS** | **CN** | **15IgAN;15IgAV-N;15HC** | **8.2;8.4;9.5** | **8(53.3%);9(60%);7(46.7%)** | **serum** | **HAA** | **U/ml** |
| **12[22]** | **2021** | **H.-X.L** | **CN** | **68IgAN;27MCD-IgAN;32HC** | **35.7;30.6;NA** | **39(57.4%);17(63%);NA** | **plasma** | **KM55** | **μg/ml** |
| **13[23]** | **2021** | **Z.-W** | **CN** | **13IgAN;13HC;13donors with IgA deposition;37donors without IgA deposition** | **NA;NA;49.9;51.3** | **NA;NA;5(38.5%);11(29.7%)** | **plasma** | **KM55** | **mg/ml** |
| **14[24]** | **2019** | **C.-W** | **CN** | **15IgAN;15CKD;15HC** | **37.8;36.8;24.6** | **8(53.3%);7(46.7%);10(66.7%)** | **serum** | **VVL** | **mg/mL** |
| **15[25]** | **2011** | **Roberta C** | **ITA** | **62IgAN;69HC** | **NA;NA** | **44(71%);48(70%)** | **serum** | **HAA** | **U/ml** |
| **16[26]** | **2005** | **W.-Q** | **CN** | **41IgAN;21OKD(2MN;1MPGN;5FSGS;11MCD;2LN);26HC** | **27.53;29.4;25** | **14(34.1%);7(33.3%);NA** | **serum** | **VVL** | **NA** |
| **17[27]** | **2019** | **X.-Z** | **CN** | **108IgAN;112IgAV-N;32HC;15IgAV** | **34.4;32.3;NA** | **54(50%);54(48.2%);NA** | **plasma** | **HPA** | **U/ml** |
| **18[28]** | **2021** | **Wasiak** | **PL** | **24IgAN;36IgAV-N;20HC** | **11.05;7.52;9.45** | **NA;NA;11(55%)** | **serum** | **KM55** | **ng/ml** |
| **19[29]** | **2018** | **William J.P** | **USA** | **135IgAN;76CKD;106HC** | **NA** | **NA** | **serum** | **KM55** | **U/ml** |
| **20[30]** | **2020** | **M.-M.T** | **CN** | **52IgAN;57IgAV-N;26IgAV;40HC** | **10.45;10.39;7.87;NA** | **40(76.9%);27(47.4%);11(42.3%);NA** | **serum** | **HPA** | **U/ml** |
| **21[31]** | **2008** | **Sachiko S** | **JPN** | **41IgAN;43OKD;38HC** | **32.7;53.4;31** | **20(48.8%);27(62.8%);16(42.1%)** | **serum** | **HAA** | **mg/mL** |
| **22[32]** | **2014** | **Kenji S** | **JPN** | **32IgAN;20HC** | **30.3;35.1** | **11(34.4%);12(60%)** | **serum** | **HAA** | **antibody titer%** |
| **23[33]** | **2019** | **Soumita B** | **IN** | **136IgAN;60OKD;50HC** | **31.9;36;41.8** | **96(70.6%);40(66.7%);22(44%)** | **serum** | **KM55** | **ng/ml** |
| **24[34]** | **2009** | **Xu XZ** | **CN** | **60 IgAN;20 MCD/MN with NS** | **38; 30** | **27(45%)；7(35%)** | **serum** | **VVL** | **AU/mg** |
| **25[35]** | **2021** | **Zhu MM** | **CN** | **40 IgAN; 20OKD(12 IMN+8 MCD); 20 HC** | **36; 47; 38** | **27(67.5%)；14(70%)；11(55%)** | **serum** | **KM55** | **μ/mL** |
| **26[36]** | **2021** | **Xiao Y** | **CN** | **40 IgAN; 20 MN; 20 HC** | **44.3; 53.5; 41.7** | **22(55%); 12(60%); 10(50%)** | **serum** | **KM55** | **mg/L** |
| **27[37]** | **2020** | **Motonori S** | **JPN** | **56 IgAN; 24 IgAV-N; 6 MCD** | **37.1; 44.4; NA** | **26 (46.4.%)；13 (54.2%); NA** | **serum** | **KM55** | **μg/mL** |
| **28[38]** | **2017** | **S. T** | **TR** | **44 IgAN; 11 Relatives of IgAN** | **38; 43** | **28 (64%); 6 (55%)** | **serum** | **KM55** | **ng/μL** |
| **29[39]** | **2009** | **X.-J.L** | **CN** | **63 IgAN;32 first-degree relatives of 19 patients;44 spouses of 44 patients;39HC** | **33.7; 37; 35.8; NA** | **32(50.8%);16(50%);19(43.2%);NA** | **serum** | **HAA** | **NA** |
| **30[40]** | **2011** | **Krzysztof K** | **USA** | **14 Pediatric IgAN;20 Pediatric IgAV-N;51 Pediatric Controls;25 Relatives of IgAN;29 Relatives of IgAV-N;141 Adult Controls** | **14.3;10.1;15.7; 43.0;39.2;36.6** | **10(71%);13(65%);28(55%);11(44%);12(41%);73(52%)** | **serum** | **HAA** | **U/ml** |
| **31[41]** | **1999** | **Alice C** | **UK** | **22 IgAN; 23 HC** | **43.5; 43.0** | **14 (64%); 15(65%)** | **serum** | **VVL** | **AU/ml** |
| **32[42]** | **2012** | **Francois B** | **FRA** | **97 IgAN;30 HC;30OKD (15 MN and 15 with biopsy proven nephro-arteriolosclerosis)** | **43.6; 45.7; 37.0** | **73 (75%); 20(66.7%); 20(66.7%)** | **serum** | **HAA** | **U/ml** |
| **33[43]** | **2008** | **KS B** | **UK** | **12 IgAN with 13 matched controls undergoing elective orthopedic surgery** | **39; 37** | **10 (83%); 7(54%)** | **serum** | **HAA** | **NA** |
| **34[44]** | **2018** | **P.-C** | **CN** | **52 IgAN; 26 IgA-MN** | **34.9; 43.6** | **25 (48.1); 17 (65.4)** | **serum** | **HAA** | **U/ml** |
| **35[45]** | **2019** | **K.-Z** | **CN** | **75 IgAN; 75 HC** | **39; NA** | **40(53.3%); NA** | **serum** | **KM55** | **μg/mL** |
| **36[46]** | **2018** | **Yukihiro W** | **JPN** | **111 IgAN; 18 IgAV-N; 29 LN; 28AAV; 13 MCD** | **40.3; 46.7; 40.9; 64.7; 37.6** | **62 (55.8); 6(33.3); 3(10.3) ; 15 (53.6); 7(53.8)** | **serum** | **KM55** | **μg/mL** |
| **37[47]** | **2022** | **Yuta U** | **JPN** | **47 IgAN; 50 HC; 43 OKD** | **NA** | **NA** | **serum** | **KM55** | **ng/μL** |
| **38[48]** | **2022** | **S.-Y.Z** | **CN** | **62 IgAN; 30OKD (10 MN+10 MCD+10 FSGS); 30 HC** | **39.10; 44.27; 39.03** | **32(51%); 18(60%); 16(53%)** | **serum** | **KM55** | **μg/mL** |
| **39[49]** | **2003** | **Linossier** | **FRA** | **44IgAN(22normal GBM+22 thin GBM); 22 HC** | **42; NA** | **22(50%);NA** | **serum** | **HAA** | **NA** |
| **40[8]** | **2016** | **Hitoshi S** | **JPN** | **207 IgAN;57 HC; 205 OKD** | **NA** | **NA** | **urine** | **HAA** | **units/mg** |
| **41[50]** | **2022** | **Katerina Z** | **CZ** | **30 IgAN;30 HC; 18 MN** | **49.4; 42; 58** | **18(60%); 17(56%); 12(66%)** | **serum** | **35A12** | **NA** |
| **42[51]** | **2022** | **Rui TT** | **CN** | **63 IgAN; 23 OKD** | **39； 33** | **36(57%)； 12(52%)** | **serum** | **KM55** | **μg/mL** |
| **43[52]** | **2021** | **Wu H** | **CN** | **40 IgAN; 25OKD; 20 HC** | **31.5； NA** | **15(37.5%); NA** | **serum** | **KM55** | **μg/mL** |
| **44[53]** | **2009** | **Jiang XY** | **CN** | **26 IgAN; 20 HC** | **9; NA** | **21(80%);16(80%)** | **serum** | **VVL** | **NA** |
| **45[54]** | **2019** | **Jiang RF** | **CN** | **37 IgAN; 35OKD; 10 HC** | **42; 50.6; 44** | **16(38%); 11(35%); 3(30%)** | **serum** | **HAA** | **U/ml** |
| **46[55]** | **2018** | **Cai Y** | **CN** | **29 IgAN; 18 HC** | **13.7; 11.0** | **19(65%); 14(77%)** | **serum** | **VVL** | **U** |
| **47[56]** | **2018** | **Liu SX** | **CN** | **52 IgAN(41 LeeⅠ-Ⅲ+11 Lee＞Ⅲ); 69 HC** | **36.9; 45.9** | **24(46%); 34(49%)** | **serum** | **VVL** | **NA** |
| **48[57]** | **1997** | **A. C. Allen** | **UK** | **9 IgAN; 12 HC** | **33; 38** | **6(67%); 6(50%)** | **serum** | **VVL** | **NA** |
| **49[58]** | **2017** | **Francois B** | **FRA** | **30 IgAN; 30 HC** | **NA; 45.7** | **NA; 20(66%)** | **serum** | **HAA** | **U/ml** |
| **50[59]** | **2005** | **L.-X. X** | **CN** | **40 IgAN(20 mmpIgAN + 20 fps IgAN); 20 HC** | **32.2; NA** | **19(47.5%); NA** | **serum** | **VVL** | **NA** |

IgAN, IgA nephropathy; HC, healthy control; Gd-IgA1, Galactose-Deficient IgA1; NA, not answer; OKD, other kidney diseases; IgAV, IgA vasculitis; IgAV-N, IgA vasculitis with nephritis; MCNS, minimal changed nephrotic syndrome; PGD, primary glomerula disease; MCD, minimal change nephrotic; LN, lupus nephritis; mmp, mild mesangial proliferative; fps, focal proliferative sclerosis; GBM, glomerular basement membrane; MesPGN, mesangial proliferative glomerulo nephritis; AAV, ANCA associated vasculitis; MN, membranous nephropathy; FSGS, focal segmental glomerulo sclerosis; MPGN, membrano proliferative glomerulo nephritis; MCG, mesangio capillary glomerulo nephritis.

Supplemental Table 3 Quality assessment of the 50 included studies with the Newcastle-Ottawa Scale.

| **Serial number** | **Publish year** | **First author** | **Selection/4** | **Comparability/2** | **Exposure/3** | **Total score of NOS/9** |
| --- | --- | --- | --- | --- | --- | --- |
| **1[11]** | **2012** | **Chen W** | **⭐⭐⭐** | **⭐** | **⭐⭐** | **6⭐** |
| **2[12]** | **2022** | **Tang YY** | **⭐⭐⭐** | **⭐⭐** | **⭐⭐** | **7⭐** |
| **3[13]** | **2006** | **Yan Y** | **⭐⭐⭐⭐** | **⭐** | **⭐⭐** | **7⭐** |
| **4[14]** | **2008** | **Qiu Q** | **⭐⭐⭐⭐** | **⭐⭐** | **⭐⭐** | **8⭐** |
| **5[15]** | **2022** | **Y.-Y.T** | **⭐⭐⭐** | **⭐** | **⭐⭐** | **6⭐** |
| **6[16]** | **2020** | **X.-Z.L** | **⭐⭐⭐** | **⭐⭐** | **⭐⭐** | **7⭐** |
| **7[17]** | **2012** | **Zou MS** | **⭐⭐⭐** | **⭐⭐** | **⭐⭐** | **7⭐** |
| **8[18]** | **2022** | **Xiao Y** | **⭐⭐⭐** | **⭐** | **⭐⭐** | **6⭐** |
| **9[19]** | **2002** | **Meng LQ** | **⭐⭐⭐** | **⭐⭐** | **⭐⭐** | **7⭐** |
| **10[20]** | **2003** | **Fu SX** | **⭐⭐** | **⭐⭐** | **⭐⭐** | **6⭐** |
| **11[21]** | **2012** | **Zou MS** | **⭐⭐⭐** | **⭐⭐** | **⭐⭐** | **7⭐** |
| **12[22]** | **2021** | **H.-X.L** | **⭐⭐⭐** | **⭐⭐** | **⭐⭐** | **7⭐** |
| **13[23]** | **2021** | **Z.-W** | **⭐⭐⭐⭐** | **⭐⭐** | **⭐⭐** | **8⭐** |
| **14[24]** | **2019** | **C.-W** | **⭐⭐⭐** | **⭐⭐** | **⭐⭐** | **7⭐** |
| **15[25]** | **2011** | **Roberta C** | **⭐⭐⭐⭐** | **⭐⭐** | **⭐⭐** | **8⭐** |
| **16[26]** | **2005** | **W.-Q** | **⭐⭐⭐** | **⭐⭐** | **⭐⭐** | **7⭐** |
| **17[27]** | **2019** | **X.-Z** | **⭐⭐⭐** | **⭐⭐** | **⭐⭐** | **7⭐** |
| **18[28]** | **2021** | **Wasiak** | **⭐⭐⭐⭐** | **⭐⭐** | **⭐⭐** | **8⭐** |
| **19[29]** | **2018** | **William J.P** | **⭐⭐⭐** | **⭐⭐** | **⭐⭐** | **7⭐** |
| **20[30]** | **2020** | **M.-M.T** | **⭐⭐⭐⭐** | **⭐⭐** | **⭐⭐** | **8⭐** |
| **21[31]** | **2008** | **Sachiko S** | **⭐⭐⭐** | **⭐⭐** | **⭐⭐** | **7⭐** |
| **22[32]** | **2014** | **Kenji S** | **⭐⭐⭐** | **⭐** | **⭐⭐** | **6⭐** |
| **23[33]** | **2019** | **Soumita B** | **⭐⭐⭐** | **⭐⭐** | **⭐⭐** | **7⭐** |
| **24[34]** | **2009** | **Xu XZ** | **⭐⭐⭐** | **⭐** | **⭐⭐** | **6⭐** |
| **25[35]** | **2021** | **Zhu MM** | **⭐⭐⭐⭐** | **⭐⭐** | **⭐⭐** | **8⭐** |
| **26[36]** | **2021** | **Xiao Y** | **⭐⭐⭐⭐** | **⭐⭐** | **⭐⭐** | **8⭐** |
| **27[37]** | **2020** | **Motonori S** | **⭐⭐⭐** | **⭐⭐** | **⭐⭐** | **7⭐** |
| **28[38]** | **2017** | **S. T** | **⭐⭐⭐** | **⭐⭐** | **⭐⭐** | **7⭐** |
| **29[39]** | **2009** | **X.-J.L** | **⭐⭐⭐** | **⭐⭐** | **⭐⭐** | **7⭐** |
| **30[40]** | **2011** | **Krzysztof K** | **⭐⭐⭐** | **⭐⭐** | **⭐⭐** | **7⭐** |
| **31[41]** | **1999** | **Alice C** | **⭐⭐⭐** | **⭐⭐** | **⭐⭐** | **7⭐** |
| **32[42]** | **2012** | **Francois B** | **⭐⭐⭐** | **⭐⭐** | **⭐⭐** | **7⭐** |
| **33[43]** | **2008** | **KS B** | **⭐⭐⭐** | **⭐⭐** | **⭐⭐** | **7⭐** |
| **34[44]** | **2018** | **P.-C** | **⭐⭐⭐** | **⭐** | **⭐⭐** | **6⭐** |
| **35[45]** | **2019** | **K.-Z** | **⭐⭐⭐⭐** | **⭐** | **⭐⭐** | **7⭐** |
| **36[46]** | **2018** | **Yukihiro W** | **⭐⭐⭐** | **⭐⭐** | **⭐⭐** | **7⭐** |
| **37[47]** | **2022** | **Yuta U** | **⭐⭐⭐⭐** | **⭐** | **⭐⭐** | **7⭐** |
| **38[48]** | **2022** | **S.-Y.Z** | **⭐⭐⭐⭐** | **⭐⭐** | **⭐⭐** | **8⭐** |
| **39[49]** | **2003** | **Linossier** | **⭐⭐⭐** | **⭐⭐** | **⭐⭐** | **7⭐** |
| **40[8]** | **2016** | **Hitoshi S** | **⭐⭐⭐** | **⭐** | **⭐⭐** | **6⭐** |
| **41[50]** | **2022** | **Katerina Z** | **⭐⭐⭐⭐** | **⭐** | **⭐⭐** | **7⭐** |
| **42[51]** | **2022** | **Rui TT** | **⭐⭐⭐** | **⭐⭐** | **⭐⭐** | **7⭐** |
| **43[52]** | **2021** | **Wu H** | **⭐⭐⭐⭐** | **⭐⭐** | **⭐⭐** | **8⭐** |
| **44[53]** | **2009** | **Jiang XY** | **⭐⭐⭐⭐** | **⭐⭐** | **⭐⭐** | **8⭐** |
| **45[54]** | **2019** | **Jiang RF** | **⭐⭐⭐⭐** | **⭐⭐** | **⭐⭐** | **8⭐** |
| **46[55]** | **2018** | **Cai Y** | **⭐⭐⭐⭐** | **⭐⭐** | **⭐⭐** | **8⭐** |
| **47[56]** | **2018** | **Liu SX** | **⭐⭐⭐⭐** | **⭐⭐** | **⭐⭐** | **8⭐** |
| **48[57]** | **1997** | **A. C. Allen** | **⭐⭐⭐** | **⭐⭐** | **⭐⭐** | **7⭐** |
| **49[58]** | **2017** | **Francois B** | **⭐⭐⭐⭐** | **⭐** | **⭐⭐** | **7⭐** |
| **50[59]** | **2005** | **L.-X. X** | **⭐⭐⭐** | **⭐⭐** | **⭐⭐** | **7⭐** |


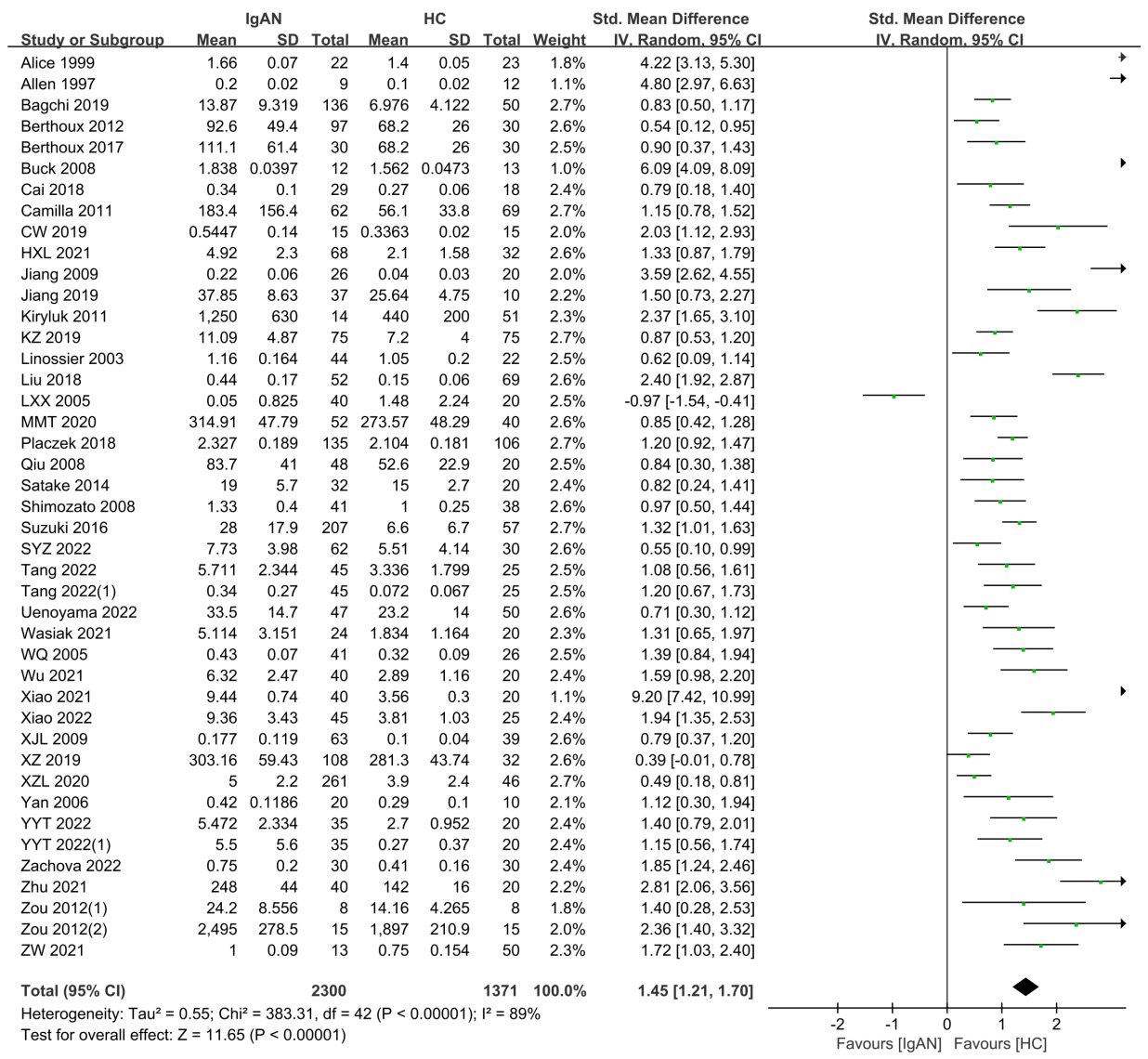


Supplemental Figure 1 The forest plot of the comparation between IgAN group and HC group.


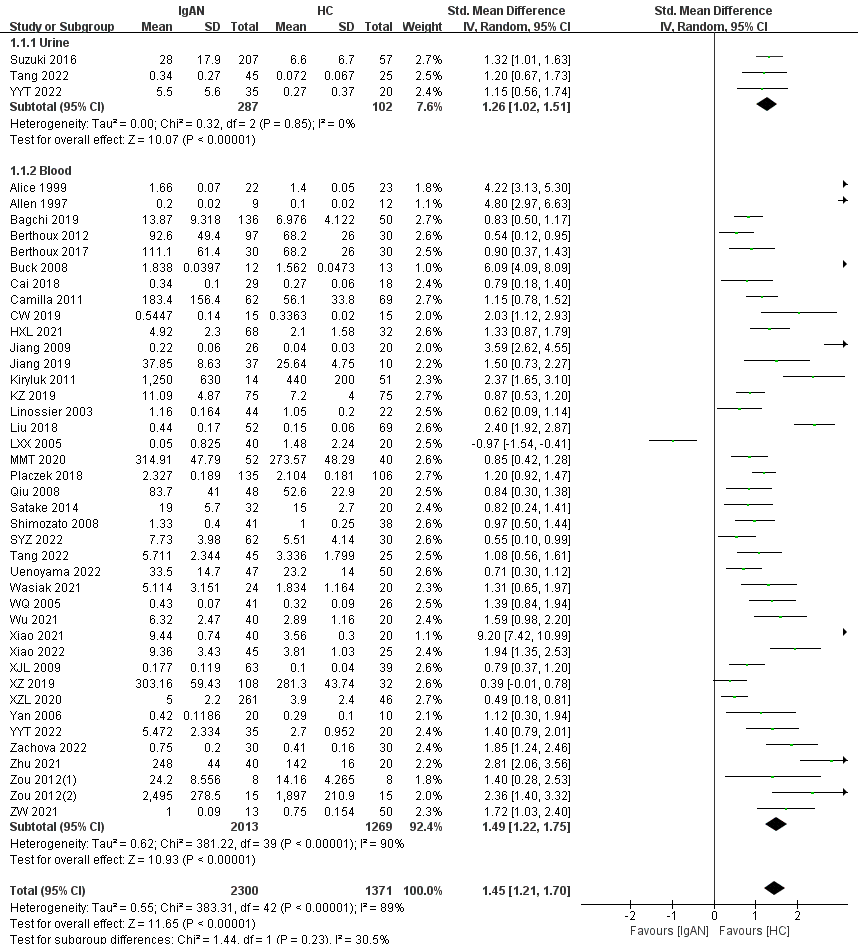


Supplemental Figure 2 The forest plot of IgAN group and HC group after subgroup analysis according to the sample types.


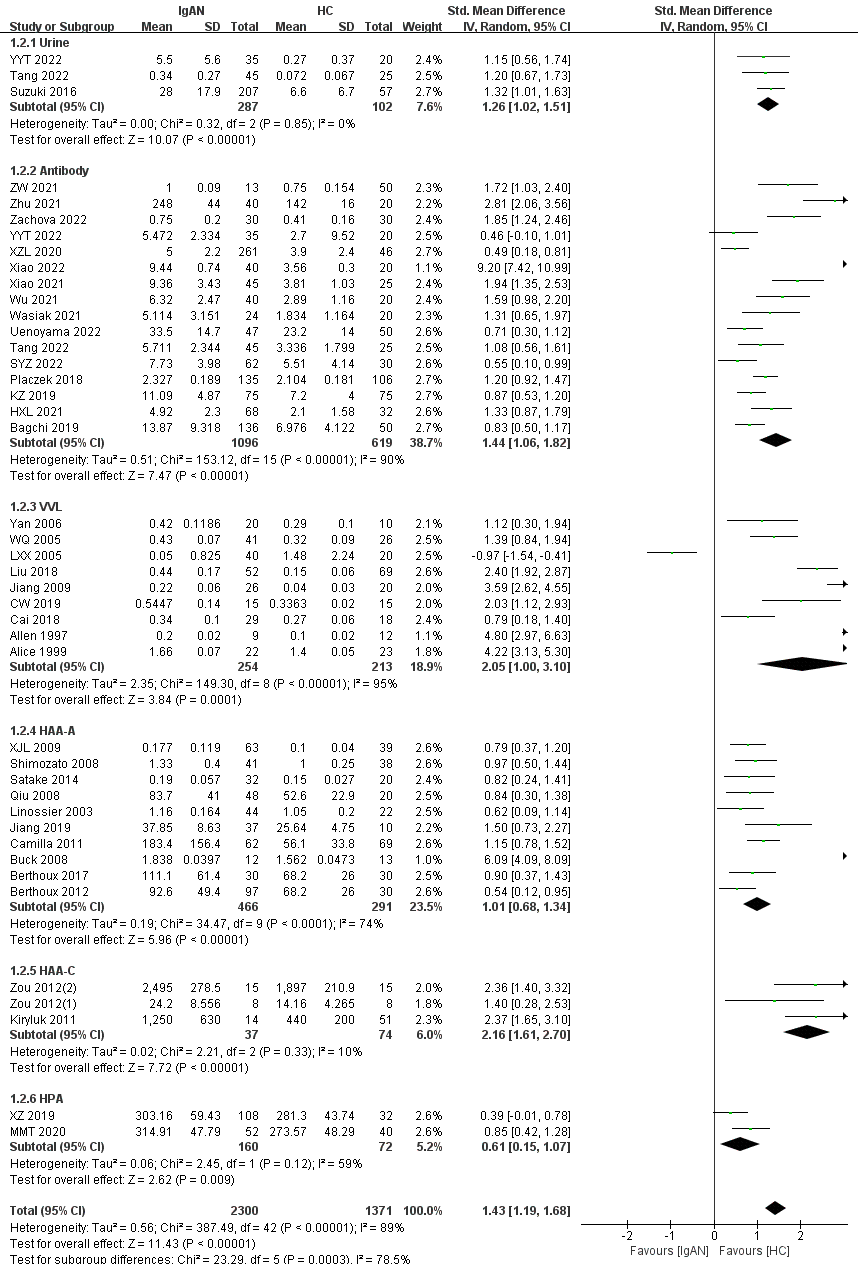


Supplemental Figure 3 The forest plot of IgAN group and HC group after subgroup analysis according to the detection methods.


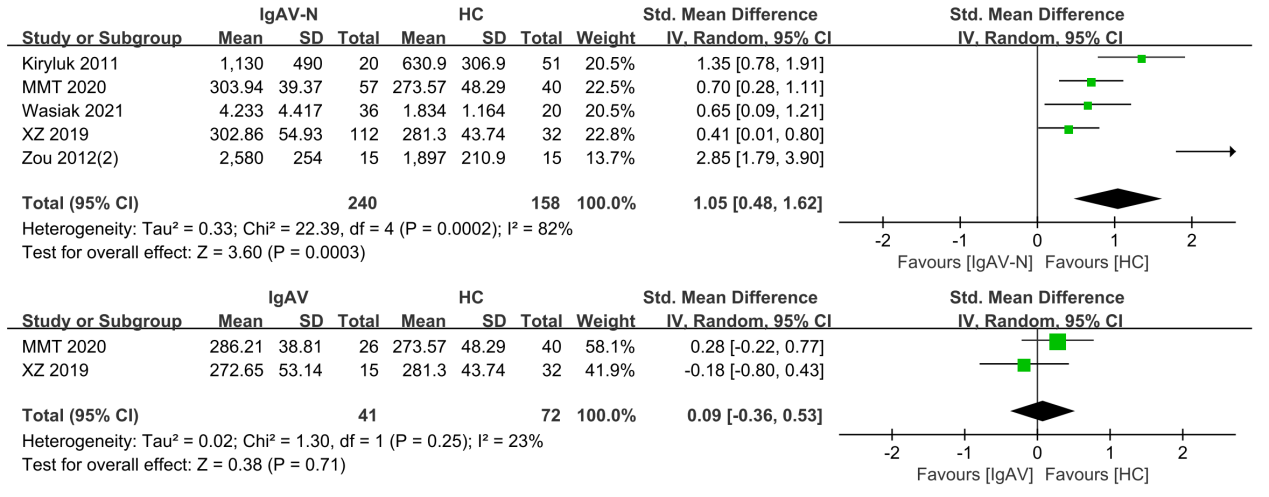


Supplemental Figure 4 The forest plot of the comparation among IgAV-N group, IgAV group and HC group.


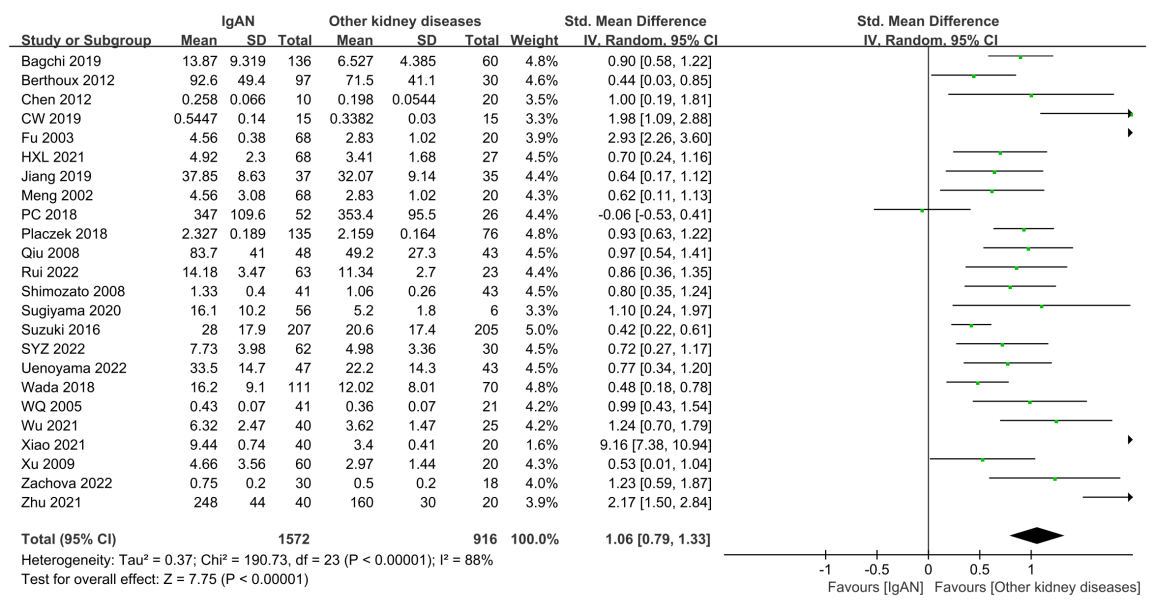


Supplemental Figure 5 The forest plots of IgAN group and other kidney diseases group.


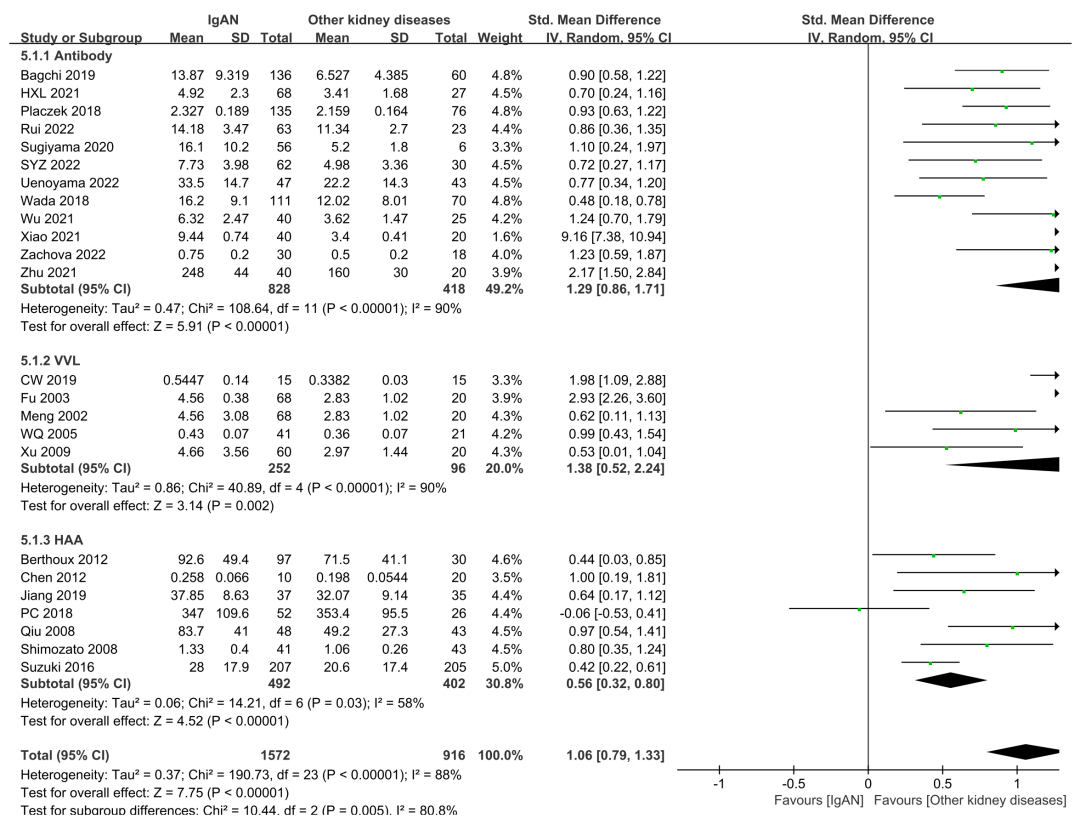


Supplemental Figure 6 The forest plots of IgAN group and other kidney diseases group after subgroup analysis.


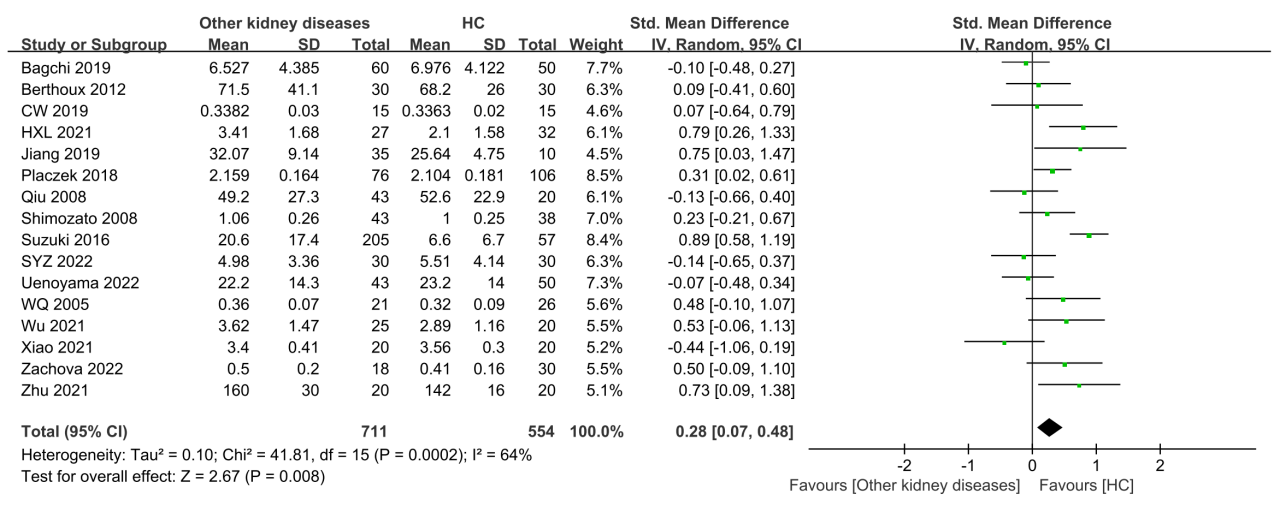


Supplemental Figure 7 The forest plots of other kidney diseases group and HC group.


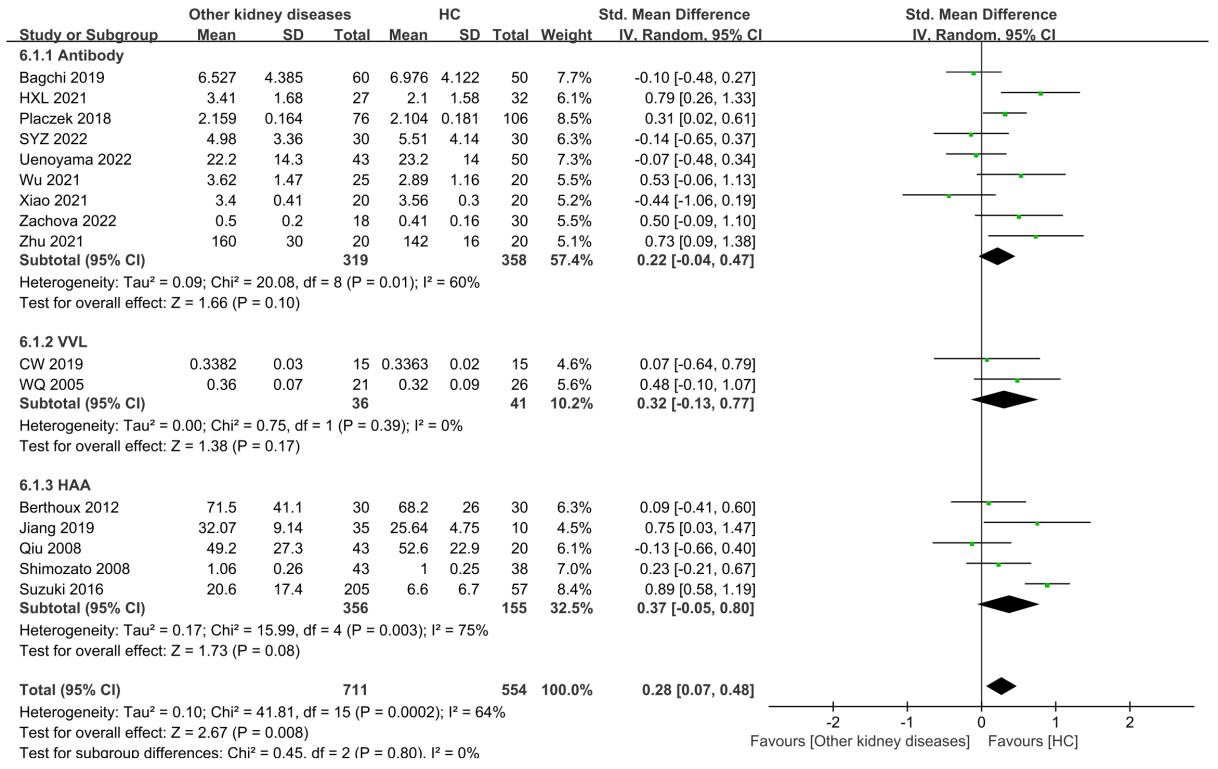


Supplemental Figure 8 The forest plots of other kidney diseases group and HC group after subgroup analysis.


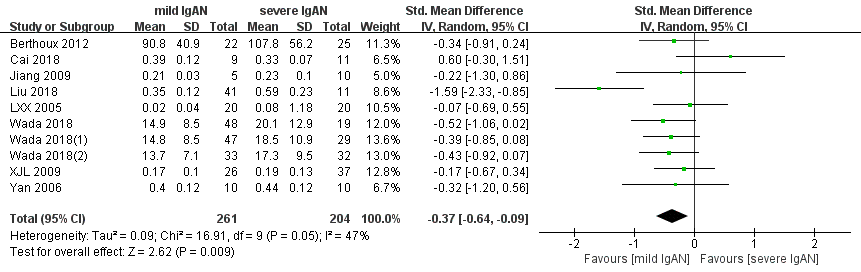


Supplemental Figure 9 The forest plots of comparison among variable grades of IgAN severity.


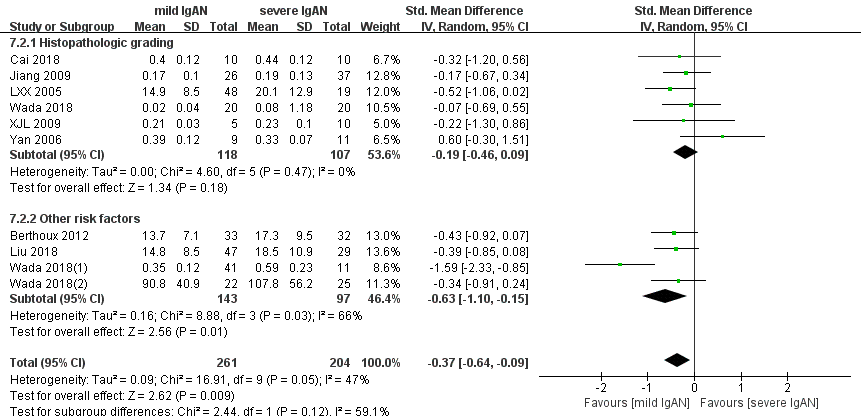


Supplemental Figure 10 The forest plots of comparison among variable grades of IgAN severity after subgroup analysis.
